# Anti-vibriosis bioactive molecules from Arctic *Penicillium* sp. Z2230

**DOI:** 10.1186/s40643-023-00628-5

**Published:** 2023-02-02

**Authors:** Jiacheng Guo, Jin Yang, Pei Wang, Bo Guo, Huifang Li, Di Zhang, Faliang An, Song Gao

**Affiliations:** 1 Jiangsu Key Laboratory of Marine Biological Resources and Environment, Jiangsu Key Laboratory of Marine Pharmaceutical Compound Screening, MNR Key Laboratory of Coastal Salt Marsh Ecosystems and Resources, School of Pharmacy, Co-Innovation Center of Jiangsu Marine Bio-Industry Technology, Jiangsu Ocean University, Lianyungang, 222005 China; 2 Key Laboratory of Bioreactor Engineering, East China University of Science and Technology, 130 Meilong Road, Shanghai, 200237 China; 3 School of Food Science and Pharmaceutical Engineering, Nanjing Normal University, Nanjing, 210023 China

**Keywords:** Arctic endophytic fungus, Secondary metabolites, Natural products, Anti-vibriosis, Aquaculture, Antibiotic resistance

## Abstract

*Vibrio* species (*Vibrio* sp.) is a class of Gram-negative aquatic bacteria that causes vibriosis in aquaculture, which have resulted in big economic losses. Utilization of antibiotics against vibriosis has brought concerns on antibiotic resistance, and it is essential to explore potential antibiotic alternatives. In this study, seven compounds (compounds **1**–**7**) were isolated from the Arctic endophytic fungus *Penicillium* sp. Z2230, among which compounds **3**, **4**, and **5** showed anti-*Vibrio* activity. The structures of the seven compounds were comprehensively elucidated, and the antibacterial mechanism of compounds **3**, **4**, and **5** was explored by molecular docking. The results suggested that the anti-*Vibrio* activity could come from inhibition of the bacterial peptide deformylase (PDF). This study discovered three *Penicillium*-derived compounds to be potential lead molecules for developing novel anti-*Vibrio* agents, and identified PDF as a promising antibacterial target. It also expanded the bioactive diversity of polar endophytic fungi by showing an example in which the secondary metabolites of a polar microbe were a good source of natural medicine.

**Graphical Abstract:**

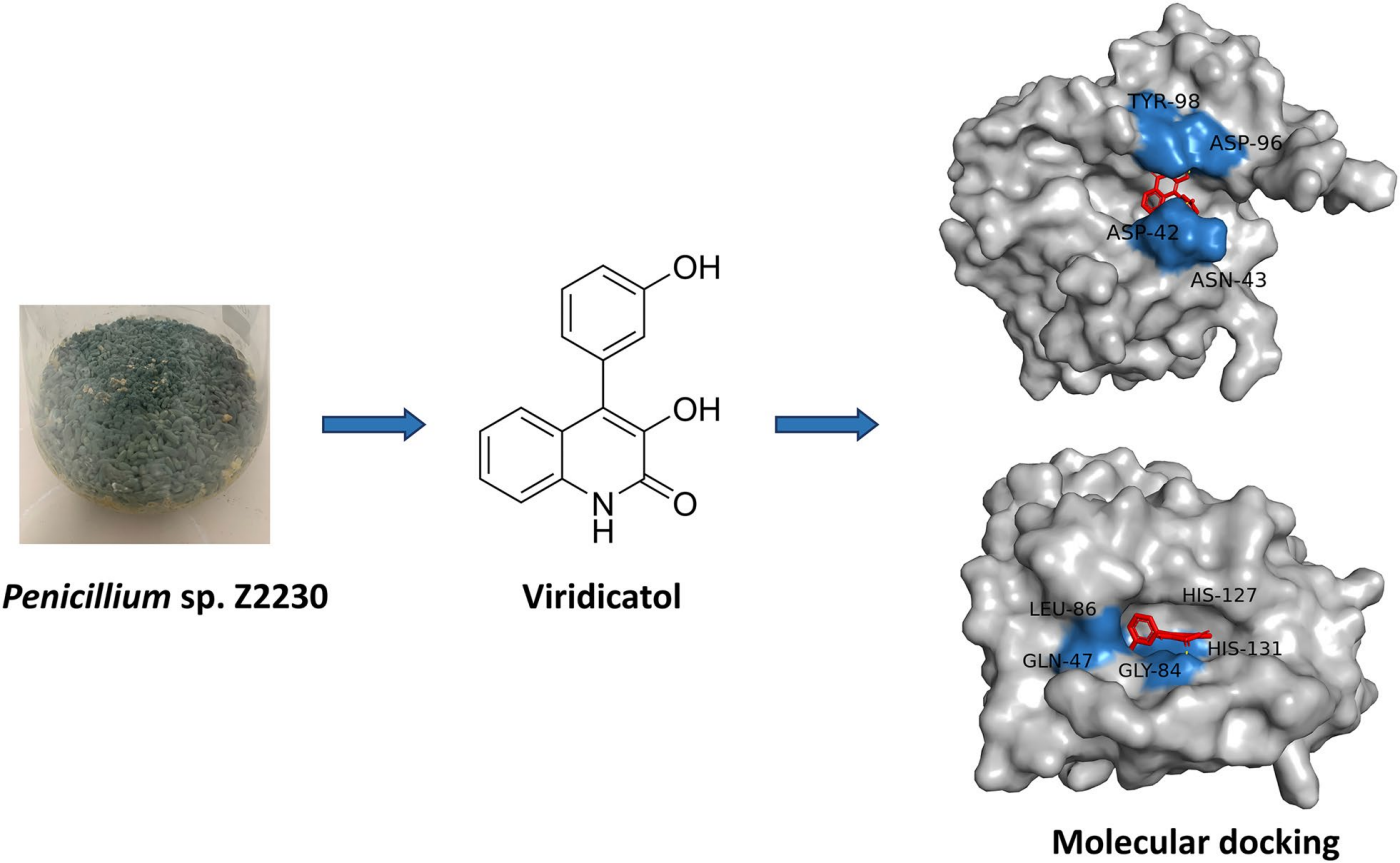

**Supplementary Information:**

The online version contains supplementary material available at 10.1186/s40643-023-00628-5.

## Introduction

*Vibrio* species (*Vibrio* sp.) are a class of Gram-negative aquatic bacteria characterized by motile rods and facultative anaerobic metabolism. They reside in estuarine and coastal environments as well as freshwaters and cause vibriosis that leads to big yield losses in aquaculture (Baker-Austin et al. 2018; Brumfield et al. 2021; Ghosh et al. 2021). *V*. *parahaemolyticus* and *V*. *alginolyticus* are the most common infectious bacteria in aquaculture (Abd El Tawab et al. 2018), while *V*. *cholerae*, *V*. *vulnificus* and *V*. *parahaemolyticus* are also serious foodborne pathogens that cause gastroenteritis, wound infections, and septicaemia in humans (Ina-Salwany et al. 2019).

In both clinical medicine and aquaculture, antibiotics like quinolone, tetracycline, and erythromycin have been used to control vibriosis (Handayani et al. 2022). With extensive usage of antibiotics, the issue of antibiotic resistance has emerged with complicated cases (Hussain et al. 2020; Li et al. 2021; Yano et al. 2014). For example, some *V*. *alginolyticus* strains in bivalves displayed resistance to vancomycin, erythromycin, and ampicillin. A strain of *V*. *campbellii* became resistant to oxytetracycline, ampicillin, and carbenicillin. And a strain of *V*. *harveyi* became resistant to more than three types of antibiotics (Kang et al. 2016). Importantly, about 64% of the *Vibrio* strains isolated from marine fishes in Southern China showed resistance to more than three antibiotic types (Nakayama et al. 2006). Alternative anti-*Vibrio* drugs are urgently needed to solve the antibiotic resistance problem.

Natural products are important sources of new drug lead compounds. Metabolites from microorganisms in extreme environments (e.g., polar regions) have become a promising source of active natural products because of the complex structural types and rich chemical diversity (Brunati et al. 2009; Robinson 2001). Particularly, polar fungi produce antimicrobial agents to kill other microbes to compete for the scarcely available nutrients in the cold and hostile environment (Gonçalves et al. 2013). It has been discovered that ketidocillinones B from Antarctica sponge-derived fungus *Penicillium* has a broad-spectrum antibacterial activity, and the ethanol extract of fungus *Purpureocillium lilacinum* displays high antimicrobial activities against a variety of bacteria and fungi (Gonçalves et al. 2015; Shah et al. 2020). Compounds from polar fungi are a good source of antibiotic alternatives.

In this study, seven compounds, 3-benzylidene-3,4-dihydro-4-methyl-H-l,4-benzodiazepine-2,5-dione (**1**), penicopeptide A (**2**), viridicatol (**3**), cyclopenol (**4**), cyclopenoin (**5**), fructigenines A (**6**), and 3-O-methylviridicatin (**7**), were isolated and determined from the Arctic endophytic fungus *Penicillium* sp. Z2230. All the compounds were tested for the antibacterial activity against four *Vibrio* species, and compounds **3**, **4**, and **5** showed distinct antibacterial activity against *V*. *parahaemolyticus*, *V*. *cholerae*, and *V*. *vulnificus*. Molecular docking studies suggested that the anti-*Vibrio* activity of compounds **3**, **4**, and **5** could come from the inhibition of bacterial peptide deformylase (PDF). These *Penicillium*-derived compounds are potential lead molecules for developing novel anti-*Vibrio* drugs.

## Materials and methods

### General experimental procedures

Optical rotations were measured using an Autopol I polarimeter (Rudolph Research Analytical Inc, Boston, MA, USA) in methanol (MeOH). UV spectra were recorded on a Shimadzu UV-1800 spectrophotometer (Shimadzu Corporation Co Ltd, Tokyo, Japan) in MeOH. 1D NMR spectra were recorded on a Bruker A VIII-600 NMR spectrometer using tetramethylsilane as an internal standard. Column chromatography was performed on silica gel (200–300 mesh, Qingdao Marine Chemical Inc, Qingdao, China) and ODS (50 µm, YMC Co Ltd, Kyoto, Japan) on a Flash Chromatograph System (SepaBen machine, Santai Technologies Inc, Changzhou, China). Preparative high-performance liquid chromatography (Pre-HPLC) was performed on a Shimadzu LC-20 system (Shimadzu Co Ltd, Tokyo, Japan) equipped with a Shim-pack RP-C18 column (20 × 250 mm, 10 µm, Shimadzu Co Ltd, Tokyo, Japan) with a flow rate of 10 mL/min at 25 °C.

### Identification of the fungus

Endophytic fungi species were isolated from the soil samples collected from Arctic Svalbard Archipelago (73–80 °N, 18–31 °E). The fungal isolates were grown in a 15-mL potato dextrose broth for 3 days at 28 °C, and the mycelia were filtered and used for DNA extraction. Fungal isolates were identified by sequencing the internal transcribed regions (ITS). Primers ITS1 (5’-TCCGTAGGTGAACCTGCGG-3’) and ITS4 (5’-TCCTCCGCTTATTGATATGC-3’) were used for sequencing the ITS of the fungal genome (Shanghai Personal Biotechnology Co Ltd, Shanghai, China). The sequencing results were aligned with fungal ITS sequences on NCBI using BLASTN. The fungus was identified to be a *Penicillium* sp. (GenBank no. OP536848) according to the sequence similarity (Schoch et al. 2012).

### Fermentation, extraction, and isolation of fungal compounds

The fungus was incubated on a potato dextrose agar (PDA) medium at 28 °C for 3 days, and the fungal mycelia were inoculated into a 250-mL Erlenmeyer flask containing 100 mL of the potato dextrose broth and fermented at 28 °C with 220 rpm. After 2 days of fermentation, the seed culture (~ 15 mL) was inoculated into an Erlenmeyer flask which contained 100 g of sterilized dry rice and 110 mL of distilled water. All the flasks were stacked at room temperature for 14 days. The fermented rice medium was extracted for three times using ethyl acetate (EtOAc), and the solvents were concentrated by a rotary evaporator to get a crude extract (43.2 g). The crude extract was loaded onto a silica gel column and eluted sequentially with a series of polar solvents, petroleum ether, dichloromethane (CH_2_Cl_2_), EtOAc, and MeOH. The EtOAc fraction was partitioned by an ODS column eluted with a gradient of MeOH–H_2_O (30–100% MeOH) and divided into five fractions, A to E. Based on TLC analysis, fractions B and E were chosen for further purification. Fraction B was purified by an ODS column (acetonitrile–H_2_O, 35:65) and a semi-preparative HPLC with 60% MeOH/H_2_O isocratic elution. Compounds **1** (8.2 mg), **2** (10.1 mg), **4** (18.6 mg), **6** (17.9 mg), and **7** (6.3 mg) were obtained from fraction B. Fraction E was purified by an ODS column (acetonitrile–H_2_O, 30:70) and a semi-preparative HPLC with 55% MeOH/H_2_O. Compounds **3** (6.1 mg) and **5** (14.5 mg) were obtained from fraction E.

### Structure determination of the compounds

The seven compounds (**1**–**7**) were dissolved in MeOH and tested with high-resolution electrospray ionization mass spectroscopy (HRESIMS) to obtain the molecular formulas. To determine the 2D structures, compounds were dissolved in deuterated chloroform (CDCl_3_) (compound **1**), deuterated methanol (MeOD) (compounds **2**, **3**, **4**, and **5**), or deuterated dimethyl sulfoxide (DMSO-*d*_6_) (compounds **6** and **7**) for NMR spectrometry. Compounds **2**, **4**, **5** and **6** were dissolved in MeOH and tested on an Autopol I polarimeter for the optical rotations.

### Anti-*Vibrio* activity

*V*. *parahaemolyticus*, *V*. *cholerae*, *V*. *vulnificus* and *V*. *alginolyticus* were cultured on 3% NaCl-LB medium (5 g/L yeast extract, 10 g/L peptone, 30 g/L NaCl, pH 7.4) plates at 30 °C for 12 h. The single colony of each strain was inoculated into a 250-mL Erlenmeyer flask containing 100 mL of the NaCl-LB liquid medium and cultured at 37 °C overnight with 200 rpm shaking. The bacterial cultures were diluted to 10^6^ CFU/mL with the NaCl-LB liquid medium for testing. Streptomycin and the seven compounds were dissolved in dimethyl sulfoxide (DMSO) to make 5-mM stocks. The stocks were diluted to desired concentrations with the NaCl-LB liquid medium for testing. Into each well of a 96-well plate, 100 μL of the 10^6^ CFU/mL bacterial dilution and 100 μL of the compound solution with the desired concentration was added. Concentrations of the compounds tested were 500, 250, 125, and 62.5 μM. The test was conducted at 30 °C overnight with three independent repeats.

### Molecular docking

Three-dimensional (3D) crystal structures or homology models of the *Vibrio*-specific targets reported in recent years on RCSB Protein Data Bank (PDB) or UniProt Database were used for molecular docking (Bonardi et al. 2021; Ding et al. 2018; Ragunathan et al. 2018; Raval et al. 2021; Sasikala et al. 2016). The AutoDock Tools 1.5.6 software was used to remove the water molecules, add the nonpolar hydrogen, and calculate the Gasteiger charges for the protein structures. Two-dimensional (2D) structures of the compounds were depicted by the ChemDraw software and transformed into PDB format through the Chem3D software as the docking ligands. In AutoDock Tools 1.5.6, the ligand was set as flexible and the receptor (the protein target) was set as rigid. The search parameters were Genetic Algorithm and the docking parameters were default. A total of 50 conformations were generated for each docking, and the conformation with the best affinity was selected as the final conformation to be visualized in the PyMOL2 software.

## Results

### Structure determination of the compounds

**Fig. 1:**
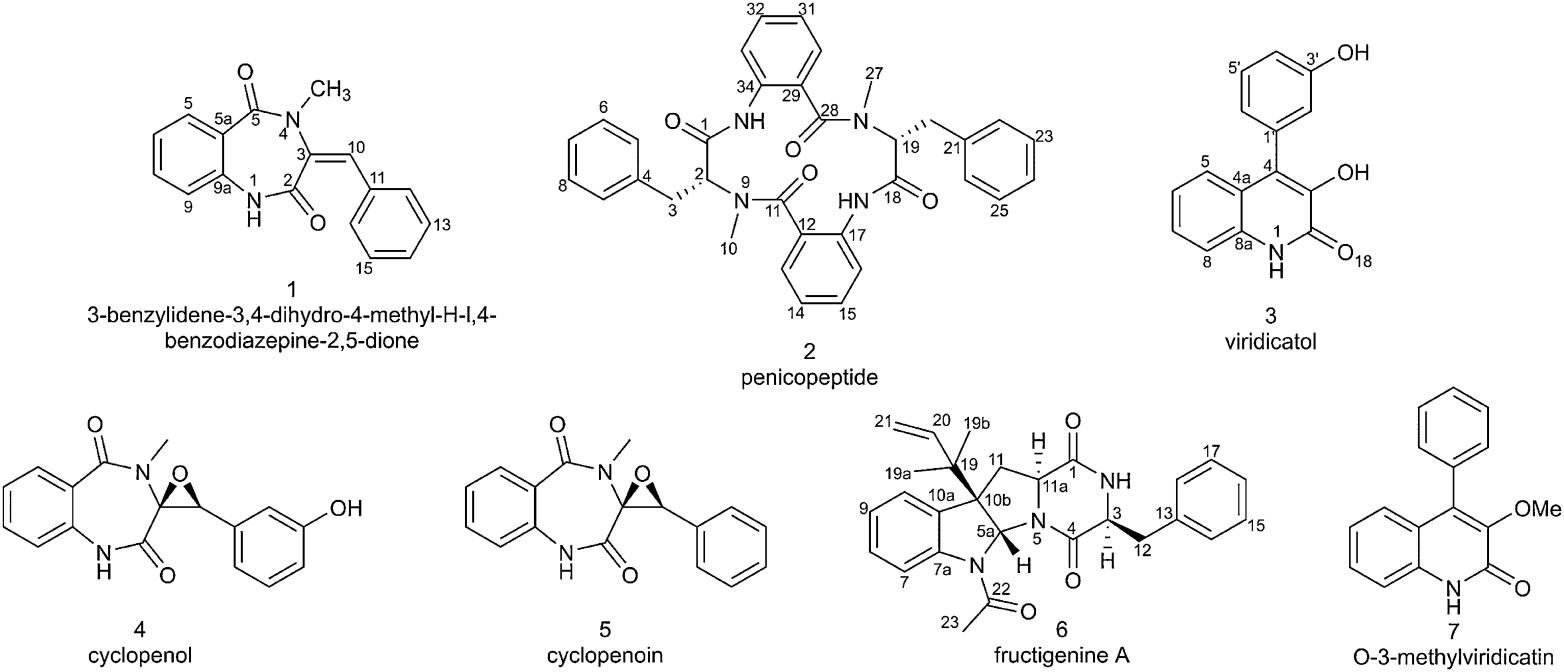
Structures of compounds isolated from *Penicillium* sp. Z2230

Seven compounds were isolated from the mycelia extracts of the Arctic endophytic fungus *Penicillium* sp. Z2230 cultured in glucose-typed rice medium. The structures were determined to be three benzodiazepines (**1**, **4**, **5**), two viridicatin derivatives (**3**, **7**), one cyclic peptide (**2**), and one diketopiperazine (**6**) (Fig. 1). For structure determination, the spectroscopic data (^1^H-NMR, ^13^C-NMR, and MS) of the seven compounds were comprehensively compared with the previous reports (Fremlin et al. 2009; Ma et al. 2017; Sun et al. 2016; Wang et al. 2020; Xin et al. 2006).

Compound **1**, yellow powder, was elucidated as 3-benzylidene-3,4-dihydro-4-methyl-H–l,4-benzodiazepine-2,5-dione with the molecular formula C_17_H_14_N_2_O_2_, which was deduced by HRESIMS with the ion peak at m/z 301.0959 [M + Na]^+^ (calcd. for C_17_H_14_N_2_O_2_Na, 301.0953). The detailed NMR data are as follows: ^1^H-NMR (400 MHz, CDCl_3_): *δ*_H_ 8.57, s, 1H (1-NH); 8.03, dd, 1H (H-12, *J* = 7.9, 1.5 Hz); 7.50, td, 1H (H-8, *J* = 7.7, 1.6 Hz); 7.43–7.34, m, 5H (H-6, H-7, H-13/15, H-16); 7.27, t, 1H (H-14, *J* = 7.6 Hz); 7.04, d, 1H (H-9, *J* = 8.0 Hz); 6.96, s, 1H (H-10); 3.20, s, 3H (4-NMe). ^13^C-NMR (150 MHz, CDCl_3_): *δ*_C_ 175.6 (C-2), 166.8 (C-5), 135.6 (C-9a), 133.4 (C-8), 132.8 (C-11), 132.1 (C-9), 131.5 (C-14), 131.5 (C-3), 129.9 (C-10), 129.5 (C-13/15), 129.1 (C-12/16), 125.6 (C-5a), 125.2 (C-7), 120.5 (C-6), 35.9 (4-NMe) (Additional file 1: Figs. S1–S4) (Sun et al. 2016).

Compound **2**, yellowish powder, was determined to be penicopeptide A with the molecular formula C_34_H_31_N_4_O_4_, which was deduced from the HRESIMS ion peak at m/z 583.2329 [M + Na]^+^ (calcd. for C_34_H_31_N_4_O_4_Na, 583.2321). The optical rotation of compound **2** was [α]20 D-26.0 (c 1.0, MeOH). The detailed NMR data are as follows: ^1^H-NMR (400 MHz, CH_3_OD): *δ*_H_ 7.98, d, 1H (H-13, *J* = 7.9 Hz); 7.84, d, 1H (*J* = 8.3 Hz); 7.61, t, 1H (*J* = 7.6 Hz); 7.52, t, 1H (*J* = 7.6 Hz); 7.36, t, 1H (*J* = 7.6 Hz); 7.15–7.32, overlapped, 8H (H-6–H-8, H-22–H-26); 7.09, d, 1H (H-33, *J* = 8.1 Hz); 7.02, d, 2H (H-5, H-9, *J* = 7.2 Hz); 4.44, t, 1H (H-19, *J* = 7.6 Hz); 4.35, dd, 1H (H-2, *J* = 10.6, 6.9 Hz); 3.42, dd, 1H (H-20a, J = 14.5, 7.9 Hz); 3.27, dd, 1H (H-20b, J = 14.5, 7.3 Hz); 3.09, s, 3H (H-27); 2.92, s, 3H (H-10); 2.81, dd, 1H (H-3a, *J* = 13.6, 6.9 Hz); 2.68, dd, 1H (H-3b, *J* = 13.6, 10.6 Hz). ^13^C-NMR (150 MHz, CH_3_OD): *δ*_C_ 170.9 (C-1), 169.8 (C-18), 169.3 (C-11), 166.9 (C-28), 136.8 (C-4), 136.6 (C-21),135.8 (C-17), 135.6 (C-34), 132.8 (C-15), 132.4 (C-32), 131.0 (C-13), 130.5 (C-30), 128.7 (C-5/7, C-22/26), 128.7 (C-6/8), 128.5 (C-23/25), 128.3 (C-7/24), 126.9 (C-14), 126.5 (C-31), 124.6 (C-12), 124.5 (C-29), 120.7 (C-16), 120.3 (C-33), 68.4 (C-2), 56.5 (C-19), 38.4 (10-NMe), 33.8 (C-3), 31.6 (C-20), 28.2 (27-NMe) (Additional file 1: Figs. S5–S8) (Sun et al. 2016).

Compound **3**, brown powder, was determined to be viridicatol with the molecular formula C_15_H_11_NO_3_, which was deduced from the HRESIMS ion peak at m/z 276.0641 [M + Na]^+^ (calcd. for C_15_H_11_NO_3_Na, 276.0637). The detailed NMR data are as follows: ^1^H-NMR (400 MHz, CH_3_OD): *δ*_H_ 7.39–7.30, m, 3H (H-5, H-5’, H-8); 7.26, d, 1H (H-6, *J* = 8.1 Hz); 7.14, dq, 1H (H-7, *J* = 8.3, 4.5, 4.1 Hz); 6.89, dd, 1H (H-4’, *J* = 7.5, 2.2 Hz); 6.86–6.80, m, 2H (H-2’, H-6’). ^13^C-NMR (150 MHz, CH_3_OD): *δ*_C_ 159.1 (C-2), 157.1 (C-3’), 141.7 (C-8a), 134.8 (C-1’), 133.0 (C-4), 129.2 (C-5’), 126.6 (C-5), 125.9 (C-7), 125.0 (C-3), 122.4 (C-6), 121.6 (C-4a), 120.8 (C-6’), 116.5 (C-2’), 115.1 (C-8), 114.6 (C-4’) (Additional file 1: Figs. S9–S12) (Ma et al. 2017).

Compound **4**, yellow powder, and was determined to be cyclopenol with the molecular formula C_17_H_14_N_2_O_4_, which was deduced from the HRESIMS ion peak at m/z 311.1037 [M + H]^+^ (calcd. for C_17_H_15_N_2_O_4_, 311.1032). The optical rotation of compound **4** was [α]20 D-97.5 (c 0.8, MeOH). The detailed NMR data are as follows: ^1^H-NMR (400 MHz, CH_3_OD) *δ*_H_ 7.59, t, 1H (H-8, *J* = 7.9 Hz); 7.19, m, 3H (H-6, H-7, H-9); 7.03, t, 1H (H-15, *J* = 7.9 Hz); 6.73, d, 1H (H-14, *J* = 7.7 Hz); 6.17, s, 1H (H-12); 6.13, d, 1H (H-16, *J* = 7.7 Hz); 4.10, s, 1H (H-10); 3.22, s, 3H (4-NMe). ^13^C-NMR (150 MHz, CH_3_OD): *δ*_C_ 167.3 (C-2), 167.1 (C-3), 157.1 (C-13), 135.1 (C-9a), 132.6 (C-8), 130.8 (C-6), 128.9 (C-15), 126.6 (C-5a), 124.7 (C-7), 121.0 (C-9), 117.1 (C-10), 115.7 (C14), 112.5 (C-12), 70.3 (C-3), 64.6 (C-10), 30.2 (4-NMe) (Additional file 1: Figs. S13–S16) (Fremlin et al. 2009).

Compound **5**, yellow powder, was determined to be cyclopenoin with the molecular formula C_17_H_14_N_2_O_3_, which was deduced from the HRESIMS ion peak at m/z 317.0905 [M + Na]^+^ (calcd. for C_17_H_14_N_2_O_3_Na, 317.0902). The optical rotation of compound **5** was [α]20 D-348.1 (c 1.0, MeOH). The detailed NMR data are as follows: ^1^H-NMR (400 MHz, CH_3_OD): *δ*_H_ 7.59, t, 1H (H-8, *J* = 7.7 Hz); 7.32, m, 1H(; 7.27–7.12, m, 4H (H-6, H-7, H-13/15); 7.08, dd, 1H (H-9, *J* = 7.9, 1.6 Hz); 6.71, d, 2H (H-12/16, *J* = 7.9 Hz, 2H); 4.20, s, 1H (H-10); 3.22, s, 3H (4-NMe). ^13^C-NMR (150 MHz, CH_3_OD): *δ*_C_ 167.2 (C-2), 167.0 (C-5), 135.2 (C-9a), 132.7 (C-8), 130.8 (C-11), 130.8 (C-9), 128.7 (C-14), 127.8 (C-13/15), 126.6 (C-5a), 125.9 (C-12/16), 124.7 (C-7), 121.0 (C-6), 70.3 (C-3), 64.6 (C-10), 30.3 (4-NMe) (Additional file 1: Figs. S17–S20) (Wang et al. 2020).

Compound **6**, yellowish powder, was determined to be fructigenine A with the molecular formula C_25_H_27_N_3_O_2_, which was deduced from the HRESIMS ion peak at m/z 402.2169 [M + H]^+^ (calcd. for C_25_H_28_N_3_O_2_, 402.2182). The optical rotation of compound **6** was [α]20 D-230.0 (c 0.6, MeOH). The detailed NMR data are as follows: ^1^H-NMR (400 MHz, DMSO- *d*_6_): *δ*_H_ 8.01, s, 1H (H-7); 7.34–7.16, m, 7H (H-8, H-10, H-14–H-18); 7.12, t, 1H (H-9, *J* = 7.5 Hz); 6.13, s, 1H (H-2); 6.03, s, 1H (H-5a); 5.74, dd, 1H (H-20, *J* = 17.2, 10.8 Hz); 5.13, d, 1H (H*cis*-21, *J* = 4.3 Hz); 5.09, d, 1H (H*trans*-21, *J* = 10.9 Hz); 4.25, dd, 1H (H-3, *J* = 9.6, 3.6 Hz); 3.77, dd, 1H (H-11a, *J* = 11.6, 5.5 Hz); 3.53–3.39, m, 1H (H-12); 2.88, dd, 1H (H-12, *J* = 14.3, 9.5 Hz); 2.66, s, 3H (H-23); 2.53, dd, 1H (H-11, *J* = 12.4, 5.6 Hz); 2.17, t, 1H (H-11, *J* = 12.0 Hz); 1.10, s, 3H (H-19b); 0.96, s, 3H (H-19a). ^13^C-NMR (150 MHz, DMSO-*d*_6_): *δ*_C_ 170.2 (C-22), 168.2 (C-4), 164.8 (C-1), 143.4 (C-7a), 143.1 (C-20), 135.4 (C-13), 132.0 (C-10a), 129.4 (C-15/17), 129.2 (C-14/18), 129.1 (C-8), 127.6 (C-16), 124.6 (C-7), 124.6 (C-10), 119.1 (C-9), 114.6 (C-21), 79.4 (C-5a), 61.0 (C-10), 59.1 (C-11a), 56.1 (C-3), 40.4 (C-19), 37.1 (C-11), 36.3 (C-12), 23.7 (C-19a), 23.2 (C-19b), 22.4 (C-23) (Additional file 1: Figs. S21–S24) (Xin et al. 2006).

Compound **7**, brown powder, was determined to be 3-O-methylviridicatin with the molecular formula C_16_H_13_NO_2_, which was deduced from the HRESIMS ion peak at m/z 274.0852 [M + Na]^+^ (calcd. for C_16_H_13_NO_2_Na, 274.0844). The detailed NMR data are as follows: ^1^H-NMR (400 MHz, DMSO- *d*_6_): *δ*_H_ 12.12, s, 1H (1-NH); 7.57–7.37, m, 5H (H-2’–H-6’); 7.35–7.31, m, 2H (H-7, H-8); 7.09, t, 1H (H-5, *J* = 7.5 Hz); 6.99, d, 1H (H-6, *J* = 8.1 Hz); 3.70, s, 3H (3-OMe). ^13^C-NMR (150 MHz, DMSO-*d*_6_): *δ*_C_ 159.0 (C-2), 145.6 (C-3), 137.9 (C-8), 136.3 (C-4), 134.0 (C-1’), 129.6 (C-2’/6’), 129.1 (C-6), 128.9 (C-3’/5’), 128.5 (C-4’), 126.2 (C-4a), 122.5 (C-5), 120.3 (C-8a), 115.6 (C-7), 59.9 (3-OMe) (Additional file 1: Figs. S25–S28) (Ma et al. 2017).

### Anti-*Vibrio* activity

**Fig. 2:**
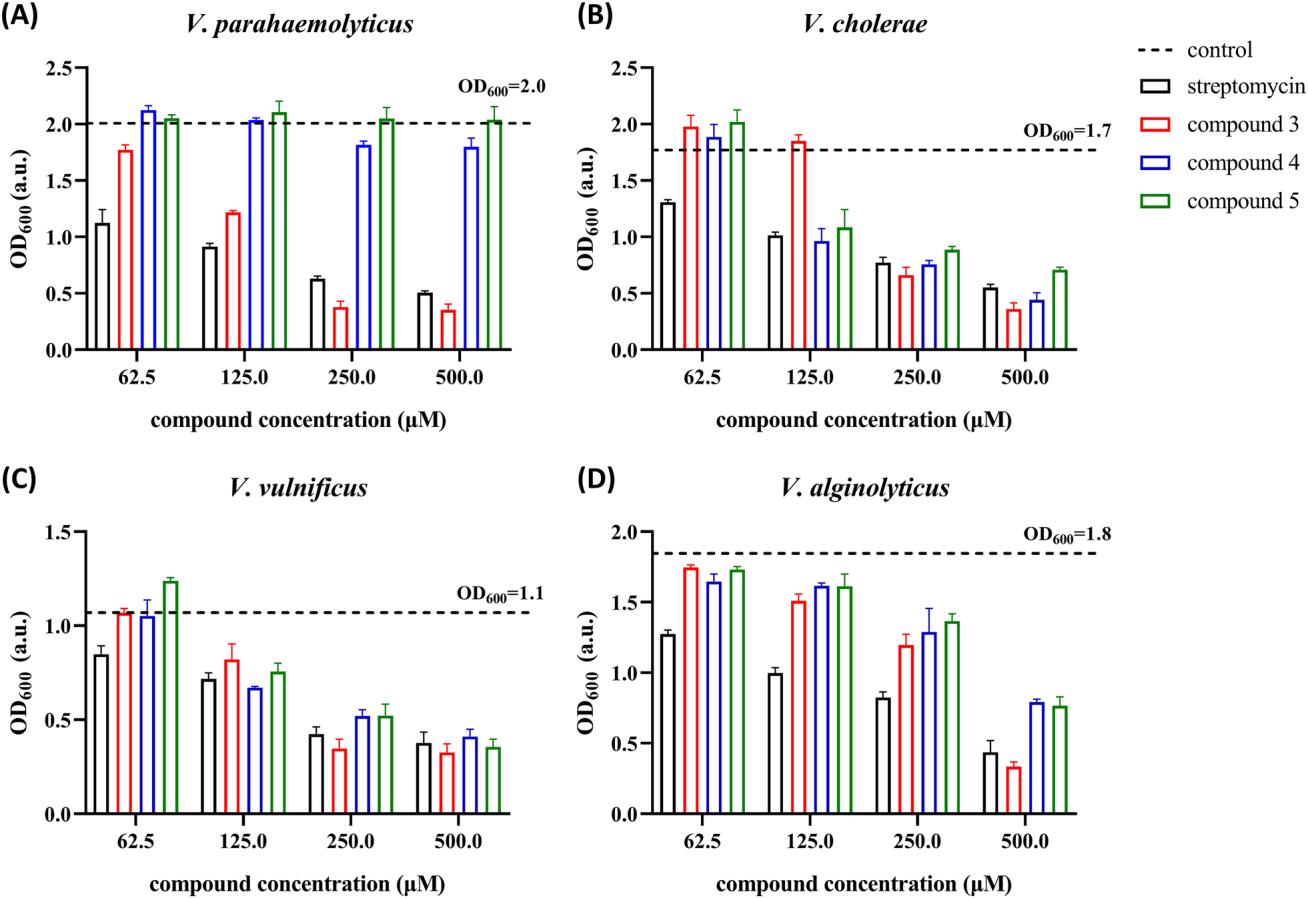
Anti-*Vibrio* activity of compounds **3**, **4** and **5**. The anti-*Vibrio* activity on *V*. *cholerae* (**A**), *V*. *parahaemolyticus* (**B**), *V*. *vulnificus* (**C**) and *V*. *alginolyticus* (**D**) is shown as bar charts. The control lines represent the OD_600_ of the control bacterial culture. The error bars represent the standard error of three parallel repeats

The anti-*Vibrio* activity of the seven compounds was tested with *V*. *parahaemolyticus*, *V*. *cholerae*, *V*. *vulnificus*, and *V*. *alginolyticus* using different concentrations of the compounds. Inhibition of bacterial cell growth by the compounds after overnight culturing was evaluated by analyzing OD_600_ of the bacterial cultures, which was related to the bacterial cell density. The OD_600_ of cultures with all the concentrations of compounds **1**, **2**, **6** and **7** had no difference comparing to the control bacterial culture (no addition of any compound), which suggested that these four compounds had no anti-*Vibrio* activity. On the other hand, compounds **3**, **4** and **5** showed anti-*Vibrio* activity. The minimum inhibitory concentration (MIC) was defined as the lowest concentration of the compound that caused the OD_600_ to be lower than the one-half of that of the control bacterial culture. In this study, the OD_600_ of the control bacterial cultures of *V*. *parahaemolyticus*, *V*. *cholerae*, *V*. *vulnificus* and *V*. *alginolyticus* were 2.0, 1.7, 1.1 and 1.8, respectively. The results showed that compound **3** had antibacterial activity against all the four *Vibrio* species, while compounds **4** and **5** had antibacterial activity against *V*. *cholerae*, *V*. *vulnificus*, and *V*. *alginolyticus* (Fig. 2). The MIC values of the seven compounds are listed in Table 1. Compounds with MIC values higher than 500 µM were considered to have no anti-*Vibrio* activity, and those MIC values were indicated as “–” in the table.

**Table 1:** The MIC of anti-*Vibrio* activities of compounds 1–7

Compound	*V*. *parahaemolyticus*	*V*. *cholerae*	*V*. *vulnificus*	*V*. *alginolyticus*
1	–	–	–	–
2	–	–	–	–
3	63.2 µg/mL (250 µM)	63.2 µg/mL (250 µM)	63.2 µg/mL (250 µM)	126.4 µg/mL (500 µM)
4	–	77.5 µg/mL (250 µM)	77.5 µg/mL (250 µM)	155.0 µg/mL (500 µM)
5	–	73.5 µg/mL (250 µM)	73.5 µg/mL (250 µM)	147.0 µg/mL (500 µM)
6	–	–	–	–
7	–	–	–	–
Streptomycin (positive)	36.3 µg/mL (125 µM)	72.6 µg/mL (250 µM)	72.6 µg/mL (250 µM)	145.2 µg/mL (500 µM)

### Study on the antibacterial mechanism

Molecular docking was used to study the antibacterial mechanism of the anti-*Vibrio* compounds **3**, **4** and **5**. *V*. *parahaemolyticus*, *V*. *cholerae*, and *V*. *vulnificus* were chosen as the models. The docking of multiple *Vibrio*-specific proteins with the three active compounds was done with the Autodock software. The target proteins were the peptide deformylases (PDF) from the three *Vibrio* species, the proline dehydrogenase (PDH) and the outer protein J (*Vop*J) from *V*. *parahaemolyticus*, the UDP-N-acetylenolpyruvoylglucosamine reductase (*Mur*B) and the carbonic anhydrase (*Vch*A) from *V*. *cholerae*, and the UDP-GlcNAC C4 epimerase (*Wbp*P) from *V*. *vulnificus*. The docking results showed that PDF had the lowest binding energy and more hydrogen bonds when docked with the three compounds (Additional file 1: Tables S1–S3). This suggested PDF could be a potential target related to the antibacterial mechanism of the three compounds.

**Fig. 3:**
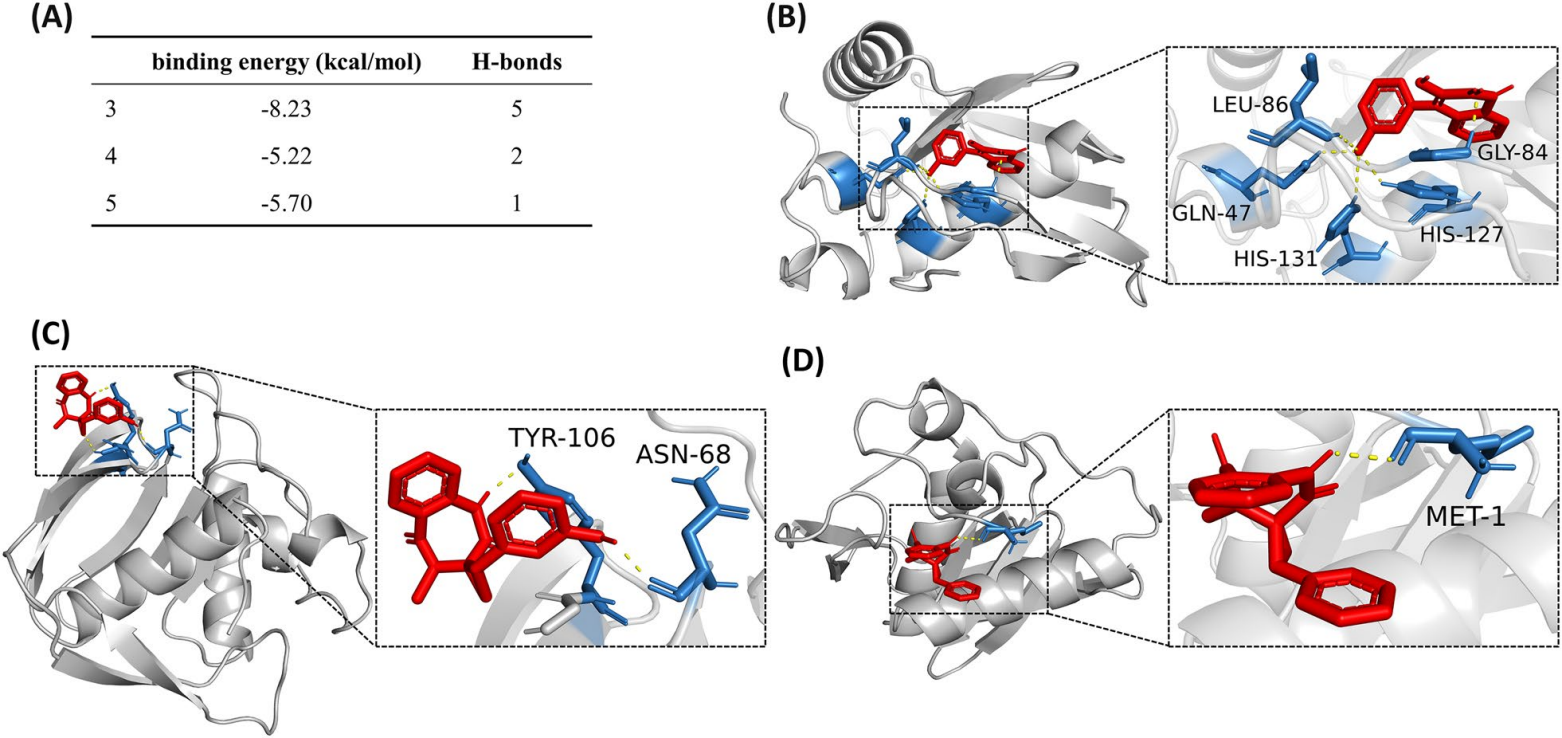
Molecular docking of compounds **3**, **4** and **5** on the PDF of *V*. *parahaemolyticus*. **A** The binding energy and number of H-bonds of the three compounds on the PDF of *V*. *parahaemolyticus*. **B**–**D** Images showing the binding pockets of the docking between compounds **3** (**B**), **4** (**C**) and **5** (**D**) and the PDF of *V*. *parahaemolyticus*

Compound **3** binds to *V*. *parahaemolyticus* PDF (PDB ID: 5mte) by interacting with five amino acid residues (Fig. 3). Among these, Gly84 forms a hydrogen bond with the carbonyl oxygen at C-2 position, while Gln47, Leu86, His127 and His131 form four hydrogen bonds with the phenolic hydroxyl at C-3’ position (Fig. 3B). The five H-bonds allows compound **3** and the target to form a stable complex with a binding energy of -8.23 kcal/mol. However, compounds **4** and **5** bind to the protein with less H-bonds and higher binding energies (-5.22 to -5.70 kcal/mol) (Fig. 3A, C and D).

**Fig. 4:**
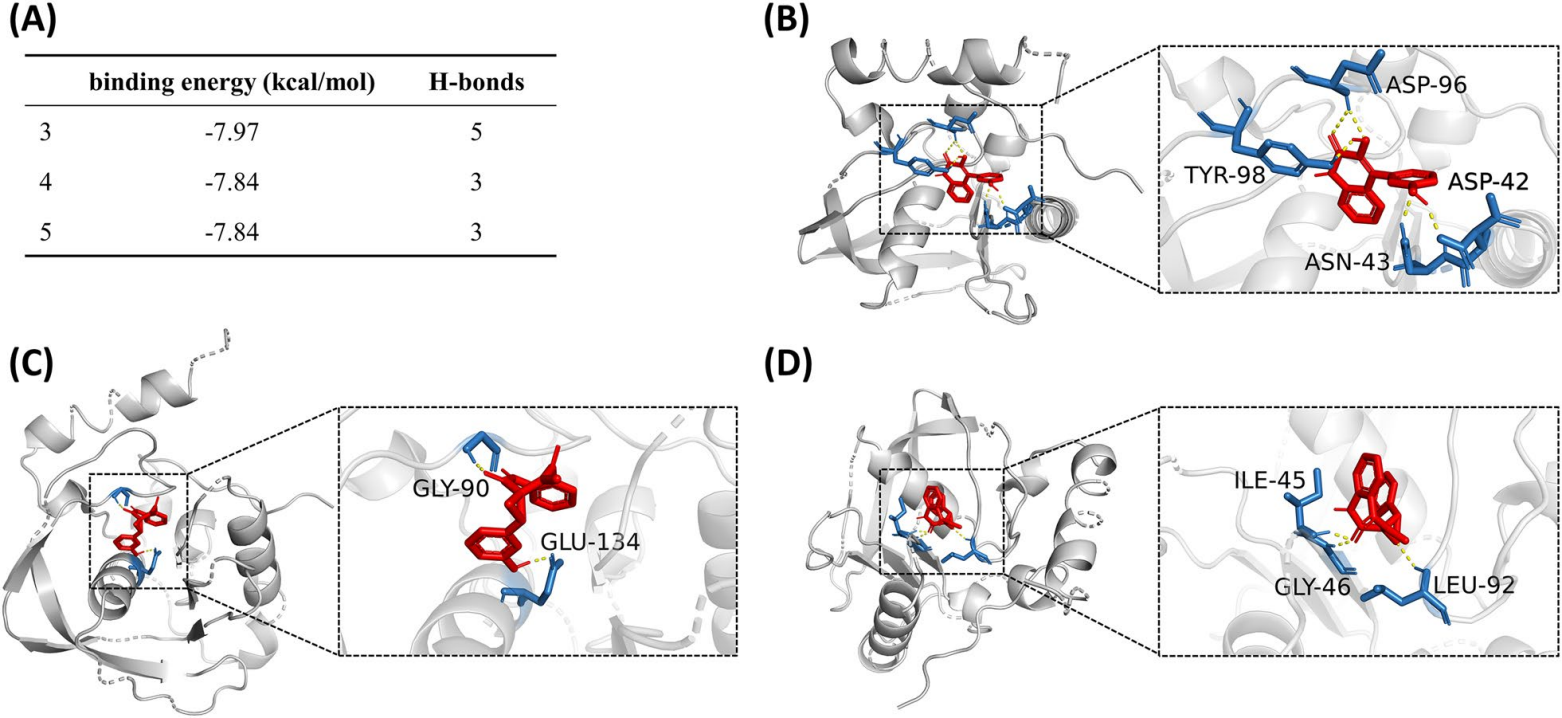
Molecular docking of compounds **3**, **4** and **5** on the PDF of *V*. *cholerae*. **A** The binding energy and number of H-bonds of the three compounds on the PDF of *V*. *cholerae*. **B**–**D** Images showing the binding pockets of the docking between compounds **3** (**B**),** 4** (**C**) and **5** (**D**) and the PDF of *V*. *cholerae*

Compound **3** forms five hydrogen bonds with a binding energy of -7.97 kcal/mol when binds to *V*. *cholerae* PDF (PDB ID: 3qu1) (Fig. 4A). Among these, Asp42 and Asn43 form two H-bonds with the phenolic hydroxyl at C-3’ position, and Asp96 forms a hydrogen bond with the carbonyl oxygen at C-2 position. Besides, the secondary amine at N-1 position forms two hydrogen bonds with Asp96 and Tyr98 (Fig. 4B). Compounds **4** and **5** exhibit the same binding energy of -7.84 kcal/mol when docked to *V*. *cholerae* PDF. Carbonyl oxygen at C-2 and hydrogen at N-1 of compound **4** form two hydrogen bonds with Gly90, and phenolic hydroxyl at C-13 forms a hydrogen bond with carbonyl oxygen of Glu134 (Fig. 4C). Compound **5** forms three hydrogen bonds with three amino acids (Ile45, Gly46 and Leu92) through C-2 carbonyl and C-3,10 epoxy groups, respectively (Fig. 4D).

**Fig. 5:**
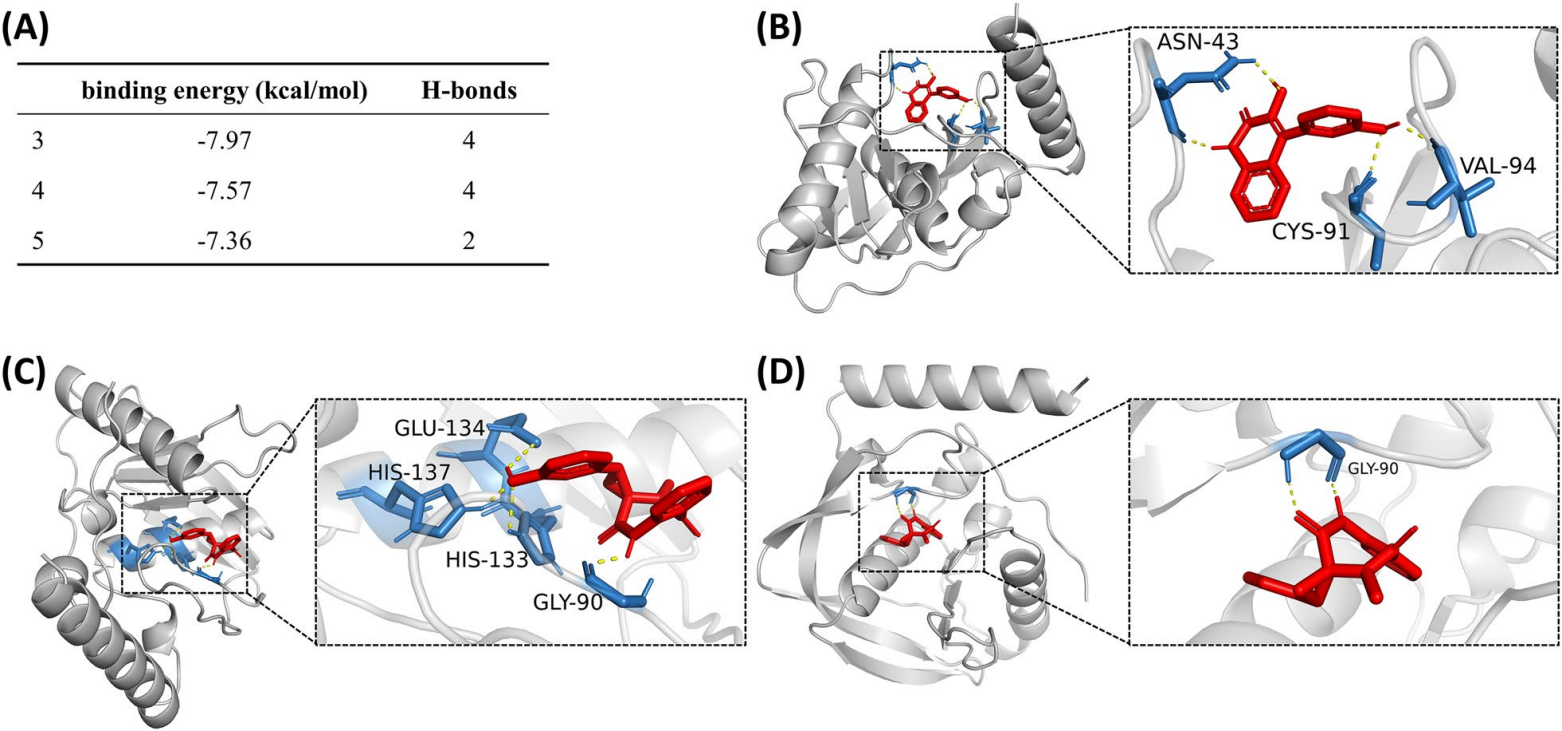
Molecular docking of compounds **3**, **4** and **5** on the PDF of *V*. *vulnificus*. **A** The binding energy and number of H-bonds of the three compounds on the PDF of *V*. *vulnificus*. **B**–**D** Images showing the binding pockets of the docking between compounds **3** (**B**),** 4** (**C**) and **5** (**D**) and the PDF of *V*. *vulnificus*

The three compounds also bind tightly to *V*. *vulnificus* PDF (Uniprot: Q597F5 (Q597F5_VIBVL)). The binding energy of compounds **3**, **4** and **5** are − 7.97, − 7.57 and − 7.36 kcal/mol, respectively (Fig. 5A). For compound **3**, Asn43 forms two hydrogen bonds with secondary amine at N-1 and alcohol hydroxyl at C-3, while Cys91 and Val94 form two hydrogen bonds with the phenolic hydroxyl at C-3’ (Fig. 5B). In the complex of compound **4** and PDF, Gly90 forms an H-bond with amino hydrogen at N-1, and His133, Glu134 and His137 form three H-bonds with phenolic hydroxyl at C-13 (Fig. 5C). Amino hydrogen at N-1 and carbonyl at C-2 of compound **5** form two H-bonds with Gly90 (Fig. 5D).

The low binding energy of these dockings showed strong binding of the compounds to the targets (PDF). Moreover, the results of these dockings are consistent with the anti-*Vibrio* activity of the compounds as shown in Table 1.

## Discussion

Microbes in extreme environments are more likely to produce secondary metabolites with complex structures and exceptional biological activities. In this study, seven compounds were isolated and determined from the Arctic-derived *Penicillium* sp. Z2230, of which compounds **3**, **4** and **5** showed particularly anti-*Vibrio* activity. This suggests that the *Penicillium* sp. from Arctic may become a solid source of active natural products.

Compounds **1**, **4** and **5** were identified as secondary metabolites of benzodiazepines, which was known as sedative-hypnotics. This study for the first time shows that benzodiazepines have anti-*Vibrio* activity. Compounds **3** and **7** were identified as viridicatin derivatives. In previous reports, viridicatin showed narrow antibacterial activity against *Escherichia coli* (Pan et al. 2017). *Vibrio* as a kind of Gram-negative bacteria can be inhibited by viridicatin derivatives as well, indicating that the viridicatin skeleton is worthy of exploring further for broad-spectrum antibacterial activities. Compound **2** was reported to have some anti-adipogenic effect, and compound **6** was reported to have certain antifungal activity (Bai et al. 2018; Sun et al. 2016). They did not show any antibacterial activity in this study.

The three active anti-*Vibrio* compounds offered a glimpse on their structure–activity relationships. For the viridicatin derivatives, compounds **3** and **7**, the anti-*Vibrio* activity of compound **3** was significantly higher than that of compound **7**, which demonstrated that the hydroxyl at C-3 and the phenyl at C-3’ were important for the activity. As shown in Table 1, the growth of *Vibrio* strains can be inhibited by compounds **4** and **5**, but not compound **1**. The ternary epoxy at C-3 on the diazepine ring of compounds **4** and **5** maybe the key group for their activities.

Recent publications have suggested that peptide deformylase (PDF) is a possible candidate of bacteriostatic target (Grzela et al. 2018). Deformylation is an essential process in microbial cells. The growth and reproduction of pathogenic microorganism will be inhibited once the sequence of PDF has mutations or deletions. The necessity of deformylation makes PDF an attractive target in developing new antibacterial agents (Meinnel and Blanquet 1993, 1994). In this study, the firm binding of the active compounds to PDF may have changed the protein conformation and affected the activity. After PDF inactivation, the formyl group of the nascent peptide chain in *Vibrio* cells could not be removed smoothly. This indicates the compounds can inhibit bacterial growth by preventing bacterial protein synthesis (Durand et al. 1999). Moreover, the result showed that electron-withdrawing epoxy units are more likely to form hydrogen bonds than π-rich double bond when binding to the ligand pockets. The discovery that PDF is present in several human parasites also suggests it to be a potential target for antiparasitic agents (Meinnel 2000).

In conclusion, seven compounds were isolated from the Arctic endophytic fungus *Penicillium* sp. Z2230. Three compounds, viridicatol **(3)**, cyclopenol **(4)** and cyclopenoin **(5)**, showed potent anti-*Vibrio* activity at micromolar level. Molecular docking of the compounds suggested that the anti-*Vibrio* activity could come from the inhibition of bacterial peptide deformylase (PDF). These *Penicillium*-derived compounds are potential lead molecules for the development of novel anti-*Vibrio* agents. The data also indicate that PDF is a promising target for new antibacterial agents. This study expands the biologically active diversity of polar endophytic fungi, and shows an example in that the secondary metabolites of polar microbes are a good source of natural medicine.

### Supplementary Information

**Additional file 1.** The detailed experiment procedures and the HRESIMS, NMR or UV spectra of compunds 1–7.

## Data Availability

All data generated or analyzed during this study are included in this research article.
