# Supplementary material for: Anti-vibriosis bioactive molecules from Arctic Penicillium sp. Z2230
Source: Bioresour Bioprocess. 2023 Feb 2;10(1):11. doi: 10.1186/s40643-023-00628-5 (PMC10992105; doi:10.1186/s40643-023-00628-5)
Supplement: Supplementary file 1 — Additional file 1. The detailed experiment procedures and the HRESIMS, NMR or UV spectra of compunds 1–7. [file 40643_2023_628_MOESM1_ESM.docx]

**Additional file 1**

**Anti-vibriosis bioactive molecules from Arctic *Penicillium* sp. Z2230**

Jiacheng Guo^1,†^, Jin Yang^2,†^, Pei Wang^3^, Bo Guo^1^, Huifang Li^1^, Di Zhang^1,*^, Faliang An^2,*^, Song Gao^1,*^

*^1^Jiangsu Key Laboratory of Marine Biological Resources and Environment, Co-Innovation Center of Jiangsu Marine Bio-industry Technology, Jiangsu Key Laboratory of Marine Pharmaceutical Compound Screening, MNR Key Laboratory of Coastal Salt Marsh Ecosystems and Resources, School of Pharmacy, Jiangsu Ocean University, Lianyungang 222005, China*

*^2^Key Laboratory of Bioreactor Engineering, East China University of Science and Technology, 130 Meilong Road, Shanghai 200237, China;*

*^3^School of Food Science and Pharmaceutical Engineering, Nanjing Normal University, Nanjing 210023, China*

^†^These authors contributed equally to this paper.

^*^Corresponding authors.

*E-mail addresses*: [dizhang@jou.edu.cn](mailto:dizhang@jou.edu.cn) (D. Zhang); [flan2016@ecust.edu.cn](mailto:flan2016@ecust.edu.cn) (F. An); [gaos@jou.edu.cn](mailto:gaos@jou.edu.cn) (S. Gao).

**Contents**

| Target alignment and screening of Molecular docking | 1-3 |
| --- | --- |
| - **Table S1** Molecular docking results of receptor proteins and compound **3** | 1 |
| - **Table S2** Molecular docking results of receptor proteins and compound **4** | 2 |
| - **Table S3** Molecular docking results of receptor proteins and compound **5** | 3 |
| Fungal materials | 4 |
| Figure S1 HRESIMS spectrum for compound **1** | 5 |
| Figure S2 UV spectrum for compound **1** | 5 |
| Figure S3 ^1^H NMR spectrum for compound **1** | 6 |
| Figure S4 ^13^C NMR spectrum for compound **1** | 6 |
| Figure S5 HRESIMS spectrum for compound **2** | 7 |
| Figure S6 UV spectrum for compound **2** | 7 |
| Figure S7 ^1^H NMR spectrum for compound **2** | 8 |
| Figure S8 ^13^C NMR spectrum for compound **2** | 8 |
| Figure S9 HRESIMS spectrum for compound **3** | 9 |
| Figure S10 UV spectrum for compound **3** | 9 |
| Figure S11 ^1^H NMR spectrum for compound **3** | 10 |
| Figure S12 ^13^C NMR spectrum for compound **3** | 10 |
| Figure S13 HRESIMS spectrum for compound **4** | 11 |
| Figure S14 UV spectrum for compound **4** | 11 |
| Figure S15 ^1^H NMR spectrum for compound **4** | 12 |
| Figure S16 ^13^C NMR spectrum for compound **4** | 12 |
| Figure S17 HRESIMS spectrum for compound **5** | 13 |
| Figure S18 UV spectrum for compound **5** | 13 |
| Figure S19 ^1^H NMR spectrum for compound **5** | 14 |
| Figure S20 ^13^C NMR spectrum for compound **5** | 14 |
| Figure S21 HRESIMS spectrum for compound **6** | 15 |
| Figure S22 UV spectrum for compound **6** | 15 |
| Figure S23 ^1^H NMR spectrum for compound **6** | 16 |
| Figure S24 ^13^C NMR spectrum for compound **6** | 16 |
| Figure S25 HRESIMS spectrum for compound **7** | 17 |
| Figure S26 UV spectrum for compound **7** | 17 |
| Figure S27 ^1^H NMR spectrum for compound **7** | 18 |
| Figure S28 ^13^C NMR spectrum for compound **7** | 18 |

**Target alignment and screening of Molecular docking**

**Table S1** Molecular docking results of receptor proteins and compound **3**

| **Docking receptors** | **H-bonds** | **Binding energy**  **(kcal/mol)** | **Residual interactions**  **(D‧‧‧H–A)** |
| --- | --- | --- | --- |
| *Vp*_PDF | 5 | -8.23 | NH(Gln47)‧‧‧O  OH(Gly84)‧‧‧O  NH(Leu86)‧‧‧O  NH(His127)‧‧‧O  NH(His131)‧‧‧O |
| *Vp*_PDH | 2 | -6.90 | OH‧‧‧O(Ala225)  NH(Lys187)‧‧‧O |
| *Vp*_VopJ | 3 | -7.64 | NH(Met159)‧‧‧O  NH(Leu201)‧‧‧O  NH(Pro206)‧‧‧O |
| *Vc*_PDF | 5 | -7.97 | OH‧‧‧O(Asp42)  NH(Asn43)‧‧‧O  OH(Asp96)‧‧‧O  OH(Asp96)‧‧‧O  OH‧‧‧O(Tyr225) |
| *Vc*_*Mur*B | 4 | -7.87 | OH‧‧‧O(Gln183)  OH‧‧‧O(Ala185)  NH‧‧‧O(Glu346)  NH(Glu346)‧‧‧O |
| *Vc*_*Vch*A | 1 | -6.66 | OH‧‧‧O(Gly58) |
| *Vv_*PDF | 4 | -7.97 | NH‧‧‧O(Asn43)  NH(Asn43)‧‧‧O  NH(Cys91)‧‧‧O  OH‧‧‧O(Val94) |
| *Vv*_*Wbp*P | 2 | -6.96 | OH‧‧‧O(Pro150)  NH‧‧‧O(His305) |

*Vp*: *V*. *parahaemolyticus*, *Vc*: *V*. *cholerae*, *Vv*: *V*. *vulnificus*.

PDF: peptide deformylase, PDH: proline dehydrogenase, *VopJ*: *V*. *parahaemolyticus*

*Mur*B: UDP-N-acetylenolpyruvoylglucosamine reductase, *Vch*A: *V*. *cholerae* carbonic anhydrase, *Wbp*P: UDP-GlcNAC C4 epimerase.

D‧‧‧H–A: donor‧‧‧hydrogen–acceptor.

**Table S2** Molecular docking results of receptor proteins and compound **4**

| **Docking receptors** | **H-bonds** | **Binding energy**  **(kcal/mol)** | **Residual interactions**  **(D‧‧‧H–A)^a^** |
| --- | --- | --- | --- |
| *Vp*_PDF | 2 | -5.22 | OH‧‧‧O(Asn68)  NH‧‧‧O(Tyr106) |
| *Vp*_PDH | 3 | -7.15 | NH(106)‧‧‧O  NH‧‧‧O(Tyr106)  OH‧‧‧O(Glu157) |
| *Vp*_*VopJ* | 2 | -6.73 | OH‧‧‧O(Glu203)  NH(Lys144)‧‧‧O |
| *Vc*_PDF | 3 | -7.84 | NH‧‧‧O(Gly90)  NH(Gly90)‧‧‧O  OH‧‧‧O(Gly134) |
| *Vc*_*Mur*B | 3 | -7.28 | OH(Arg227)‧‧‧O  OH(Asn239)‧‧‧O  NH‧‧‧O(Leu123) |
| *Vc*_*Vch*A | 3 | -5.98 | NH(Asn90)‧‧‧O  NH(Gln165)‧‧‧O  OH‧‧‧O(Ser197) |
| *Vv*_PDF | 4 | -7.57 | NH‧‧‧O(Gly90)  NH(His133)‧‧‧O  OH(Glu134)‧‧‧O  NH(His137)‧‧‧O |
| *Vv*_*Wbp*P | 3 | -7.52 | NH(Gln201)‧‧‧O  OH‧‧‧O(Asn204)  NH(Ala209)‧‧‧O |

*Vp*: *V*. *parahaemolyticus*, *Vc*: *V*. *cholerae*, *Vv*: *V*. *vulnificus*.

PDF: peptide deformylase, PDH: proline dehydrogenase, *VopJ*: *V*. *parahaemolyticus*

*Mur*B: UDP-N-acetylenolpyruvoylglucosamine reductase, *Vch*A: *V*. *cholerae* carbonic anhydrase, *Wbp*P: UDP-GlcNAC C4 epimerase.

D‧‧‧H–A: donor‧‧‧hydrogen–acceptor.

**Table S3** Molecular docking results of receptor proteins and compound **5**

| **Docking receptors** | **H-bonds** | **Binding energy**  **(kcal/mol)** | **Residual interactions**  **(D‧‧‧H–A)^a^** |
| --- | --- | --- | --- |
| *Vp*_PDF | 1 | -5.70 | NH‧‧‧O(Met1) |
| *Vp*_PDH | 2 | -6.84 | NH‧‧‧O(Lys94)  NH(Lys94)‧‧‧O |
| *Vp_VopJ* | 1 | -6.34 | NH(Lys144)‧‧‧O |
| *Vc*_PDF | 3 | -7.84 | NH(Ile45)‧‧‧O  NH(Gly46)‧‧‧O  NH(Leu92)‧‧‧O |
| *Vc*_*Mur*B | 2 | -7.33 | NH‧‧‧O(Leu123)  NH(Arg227)‧‧‧O |
| *Vc*_*Vch*A | 3 | -5.70 | NH(Asn160)‧‧‧O  NH(Gln165)‧‧‧O  OH‧‧‧O(Arg198) |
| *Vv*_PDF | 2 | -7.36 | NH‧‧‧O(Gly90)  OH(Gly90)‧‧‧O |
| *Vv*_*Wbp*P | 2 | -7.03 | NH(Glu201)‧‧‧O  NH(Ala209)‧‧‧O |

*Vp*: *V*. *parahaemolyticus*, *Vc*: *V*. *cholerae*, *Vv*: *V*. *vulnificus*.

PDF: peptide deformylase, PDH: proline dehydrogenase, *VopJ*: *V*. *parahaemolyticus*

*Mur*B: UDP-N-acetylenolpyruvoylglucosamine reductase, *Vch*A: *V*. *cholerae* carbonic anhydrase, *Wbp*P: UDP-GlcNAC C4 epimerase.

D‧‧‧H–A: donor‧‧‧hydrogen–acceptor.

**Fungal materials**

The ITS RNA sequence data for this strain was listed as follows: 5’-CCGAGGTCACCTGGATAAAAATTTGGGTTGATCGGCAAGCGCCGGCCGGGCCTACAGAGCGGGTGACAAAGCCCCATACGCTCGAGGACCGGACGCGGTGCCGCCGCTGCCTTTCGGGCCCGTCCCCCGGAATCGGAGGACGGGGCCCAACACACAAGCCGGGCTTGAGGGCAGCAATGACGCTCGGACAGGCATGCCCCCCGGAATACCAGGGGGCGCAATGTGCGTTCAAAGACTCGATGATTCACTGAATTTGCAATTCACATTACGTATCGCATTTCGCTGCGTTCTTCATCGATGCCGGAACCAAGAGATCCGTTGTTGAAAGTTTTAAATAATTTATATTTTCACTCAGACTTCAATCTTCAGACAGAGTTCGGGGGTGTCTTCGGCGGGCGCGGGCCCGGGGGCGTGAGCCCCCCGGCGGCCAGTAAAGGCGGGCCCGCCGAAGCAACAAGGTAAAATAAACACGGGTGGGAGGTTGGACCCAAAGGGCCCTCACTCGGTAATGATCCTTCCGCAGGTTCCCCTACGGA-3’. The GenBank number was OP536848.


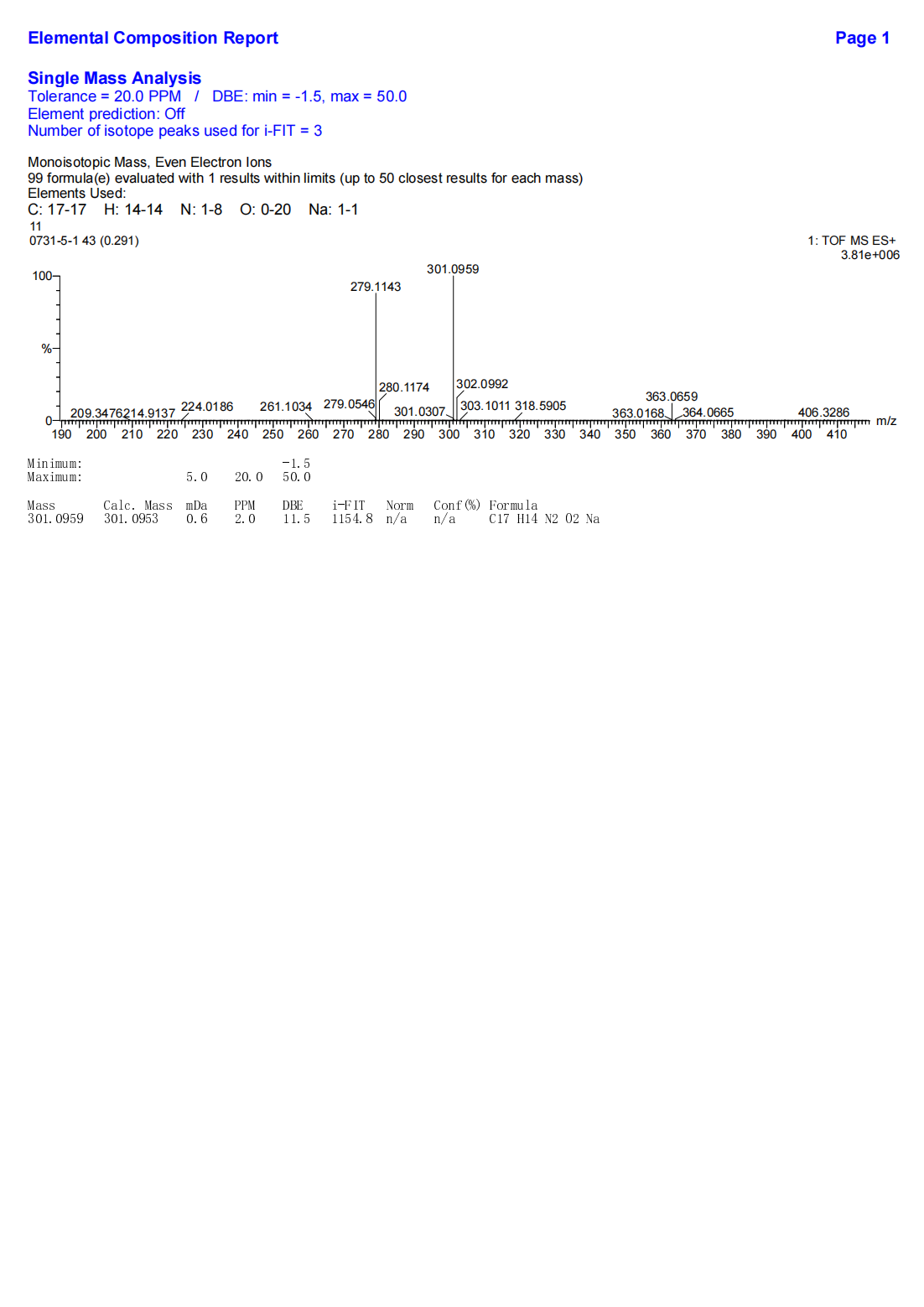


**Figure S1** HRESIMS spectrum for compound **1**

**Figure S2** UV spectrum for compound **1**


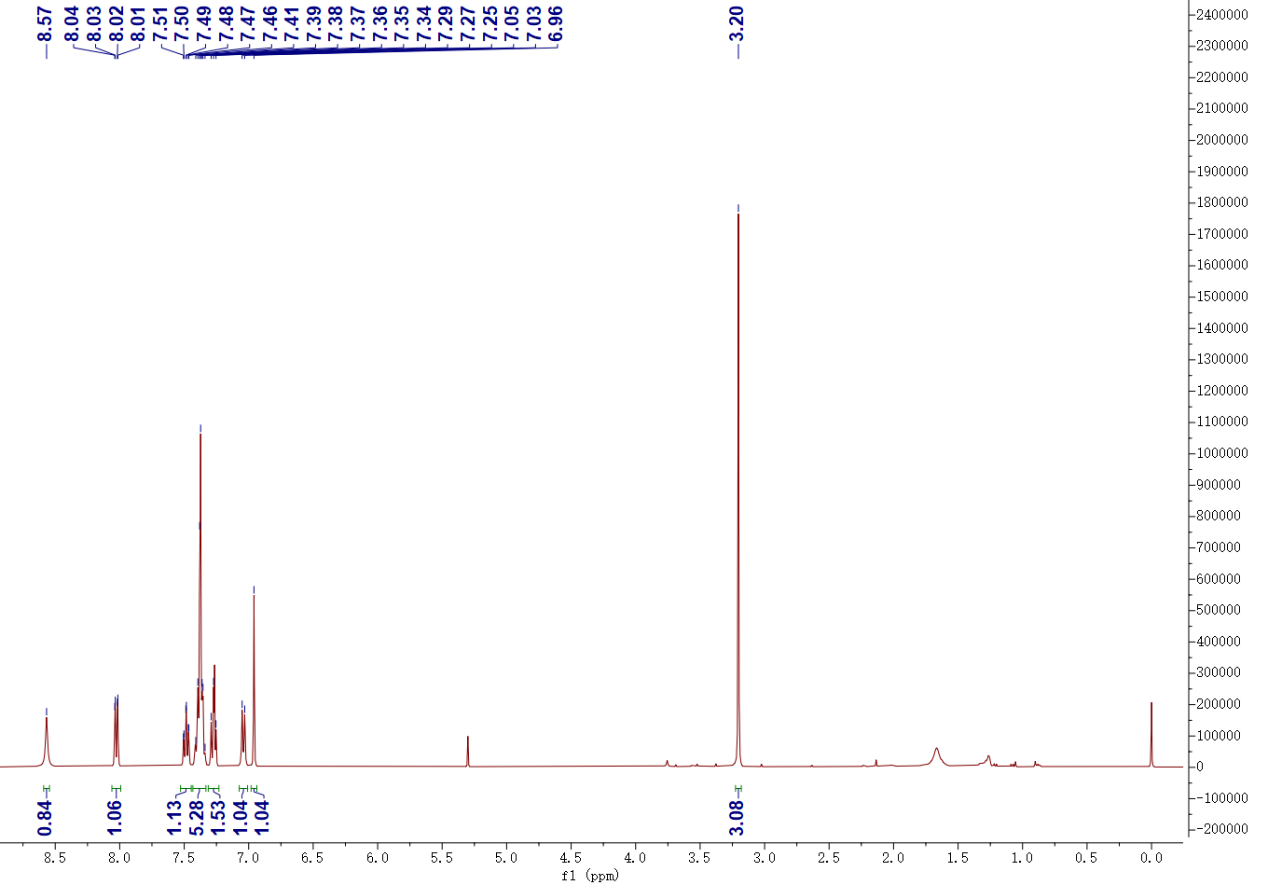


**Figure S3** ^1^H NMR spectrum for compound **1**


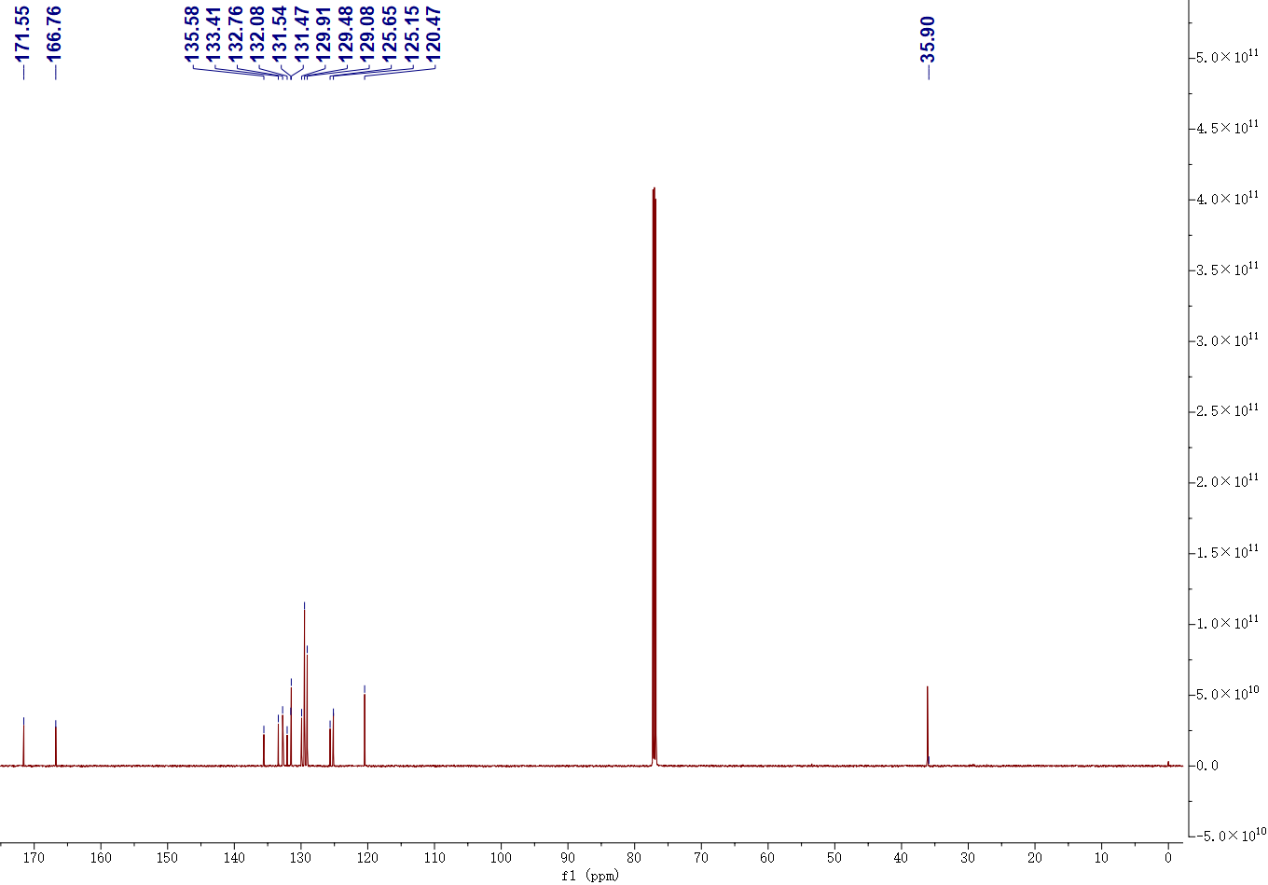


**Figure S4** ^13^C NMR spectrum for compound **1**


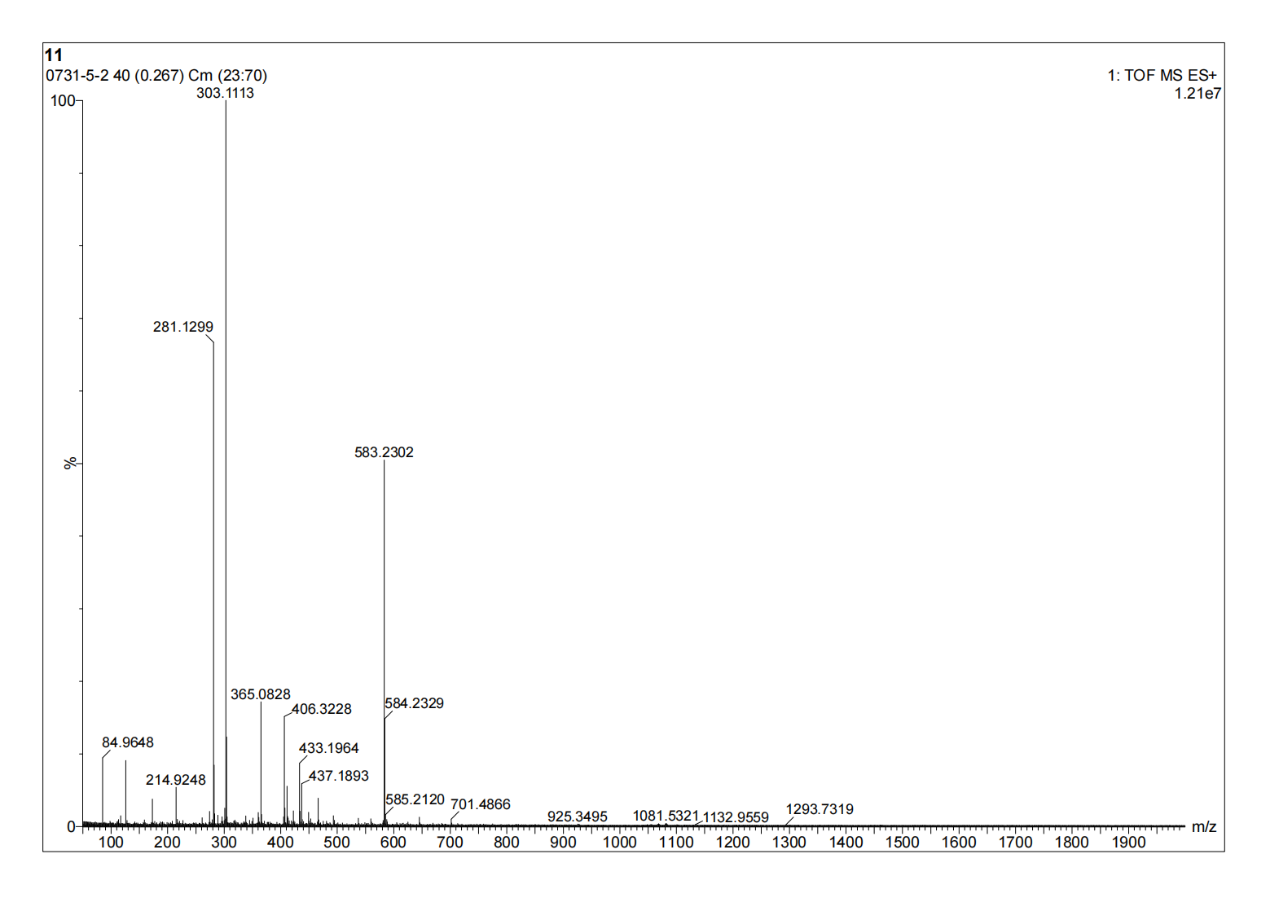


**Figure S5** HRESIMS spectrum for compound **2**

**Figure S6** UV spectrum for compound **2**


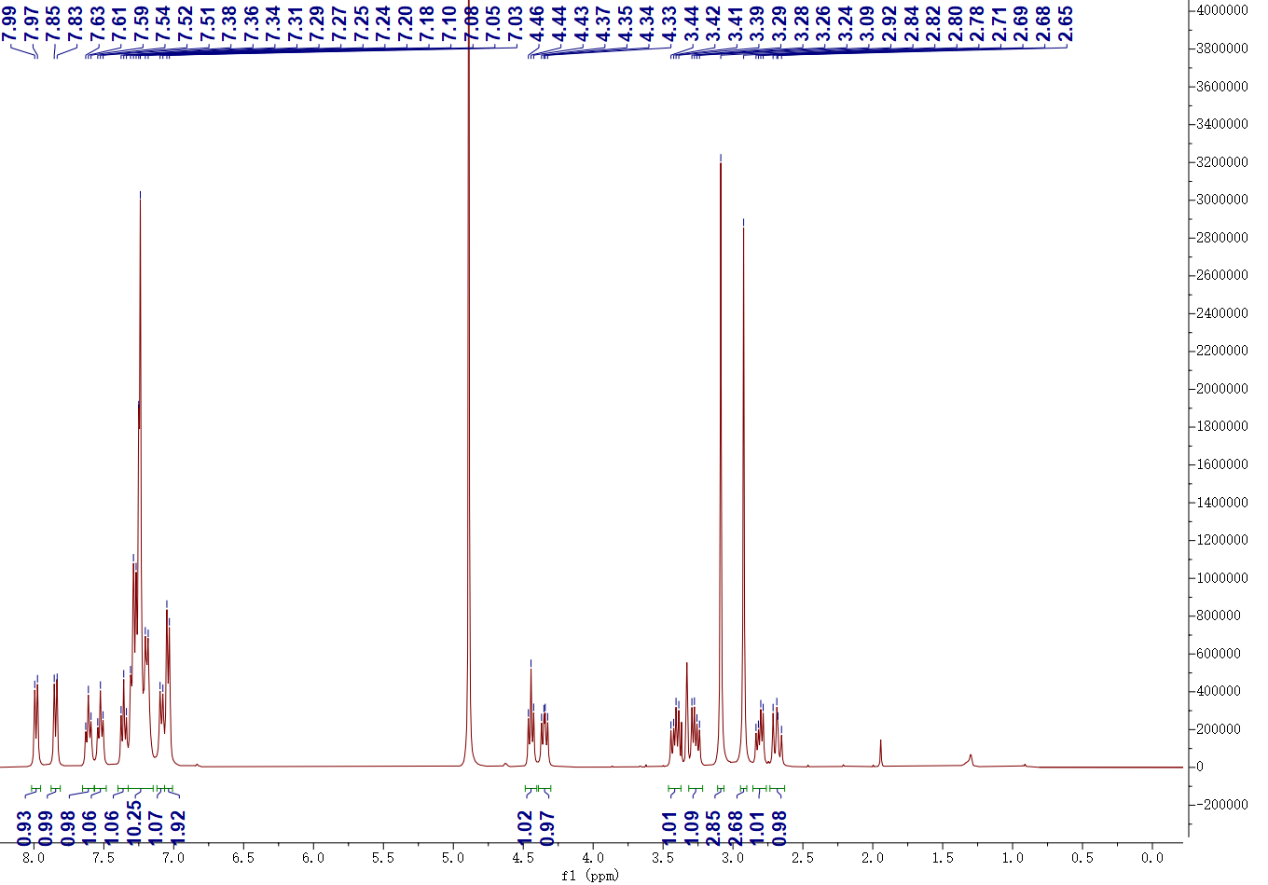


**Figure S7** ^1^H NMR spectrum for compound **2**


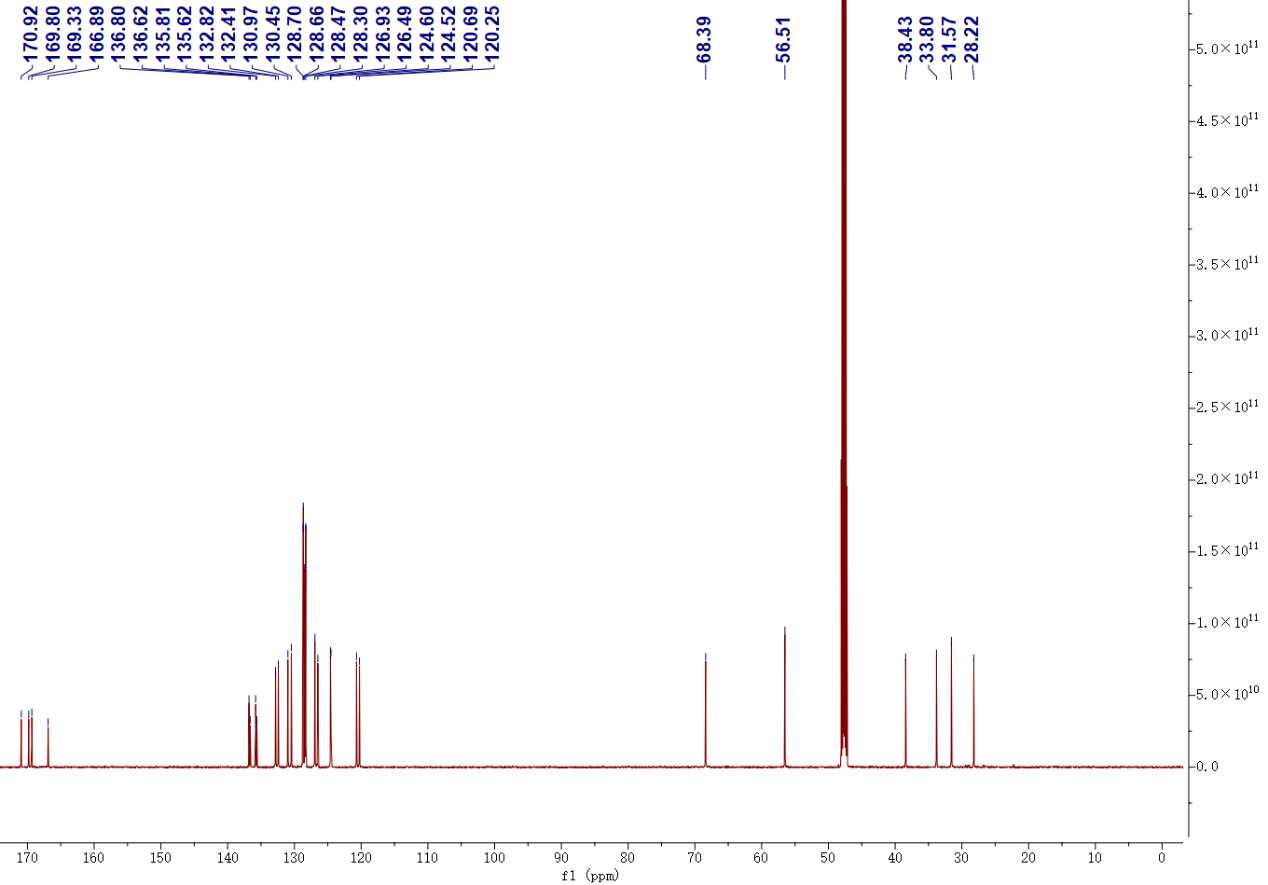


**Figure S8** ^13^C NMR spectrum for compound **2**


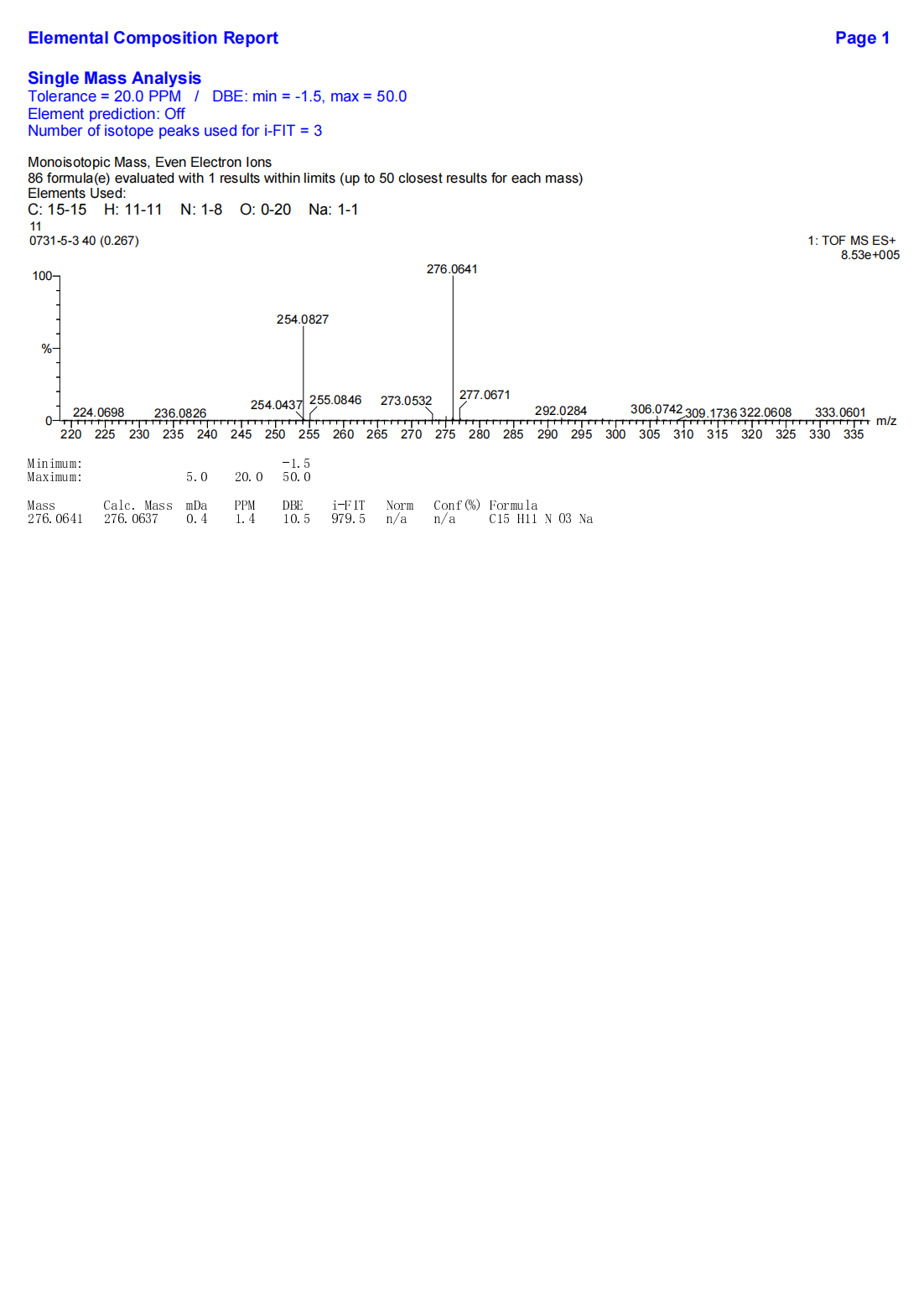


**Figure S9** HRESIMS spectrum for compound **3**

**Figure S10** UV spectrum for compound **3**


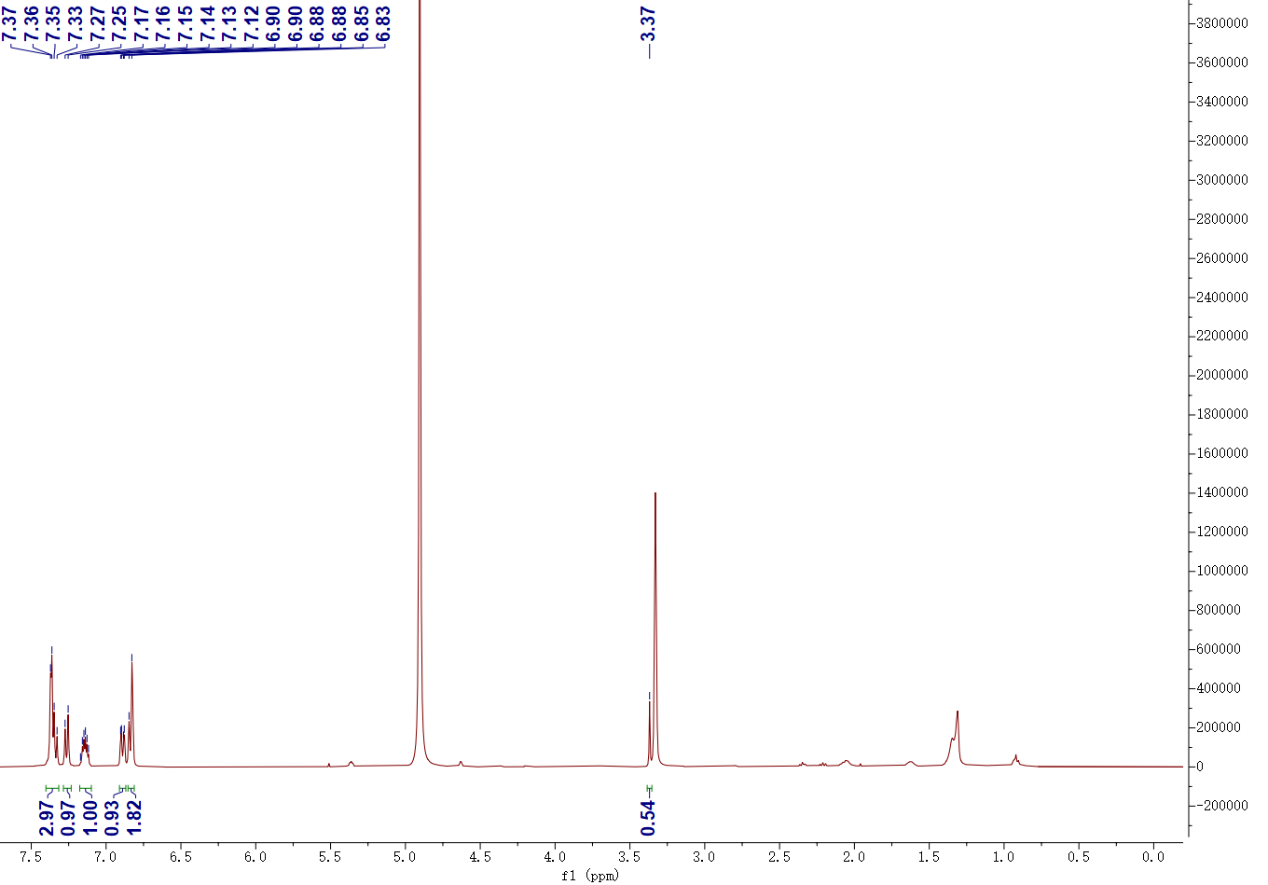


**Figure S11** ^1^H NMR spectrum for compound **3**


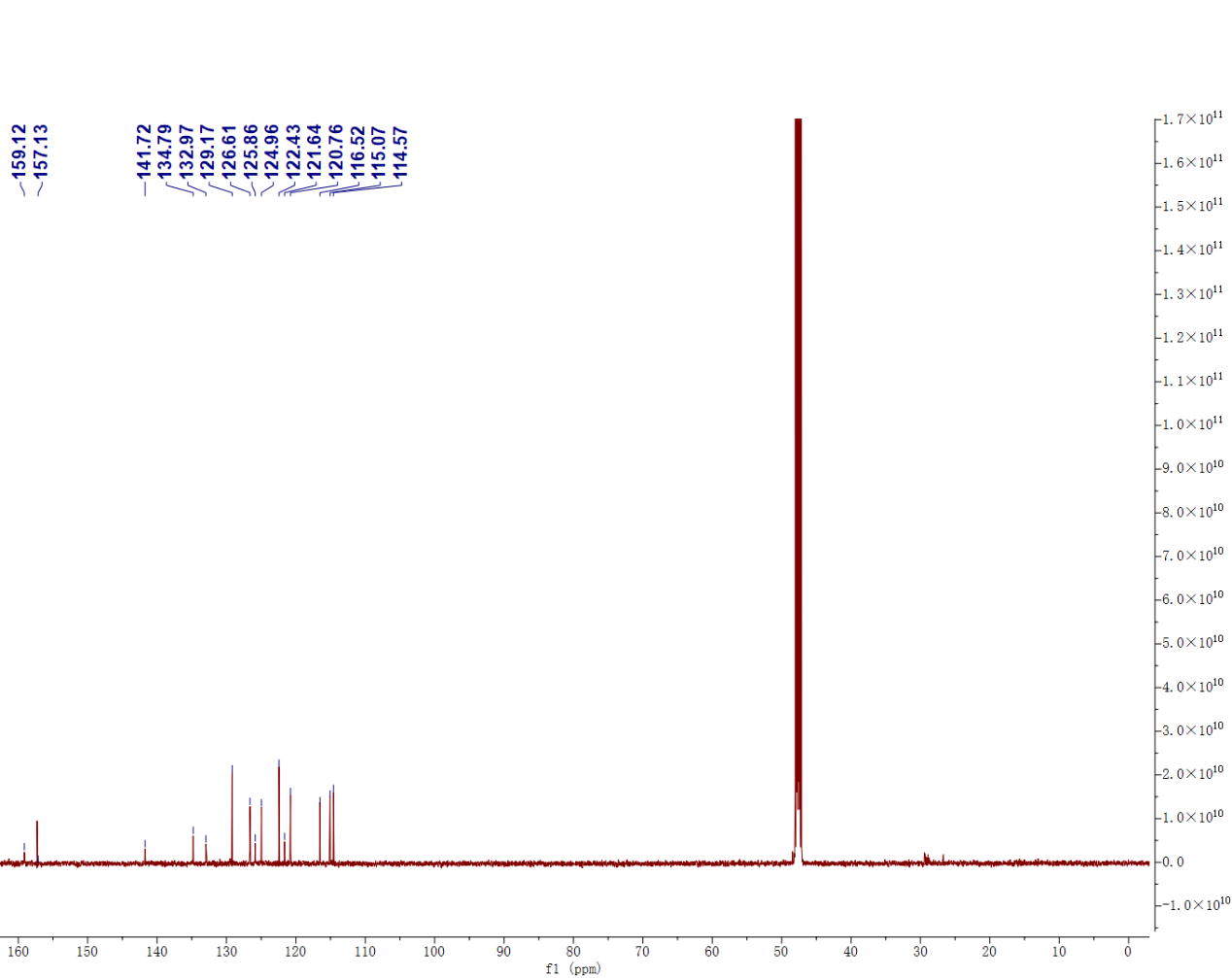


**Figure S12** ^13^C NMR spectrum for compound **3**


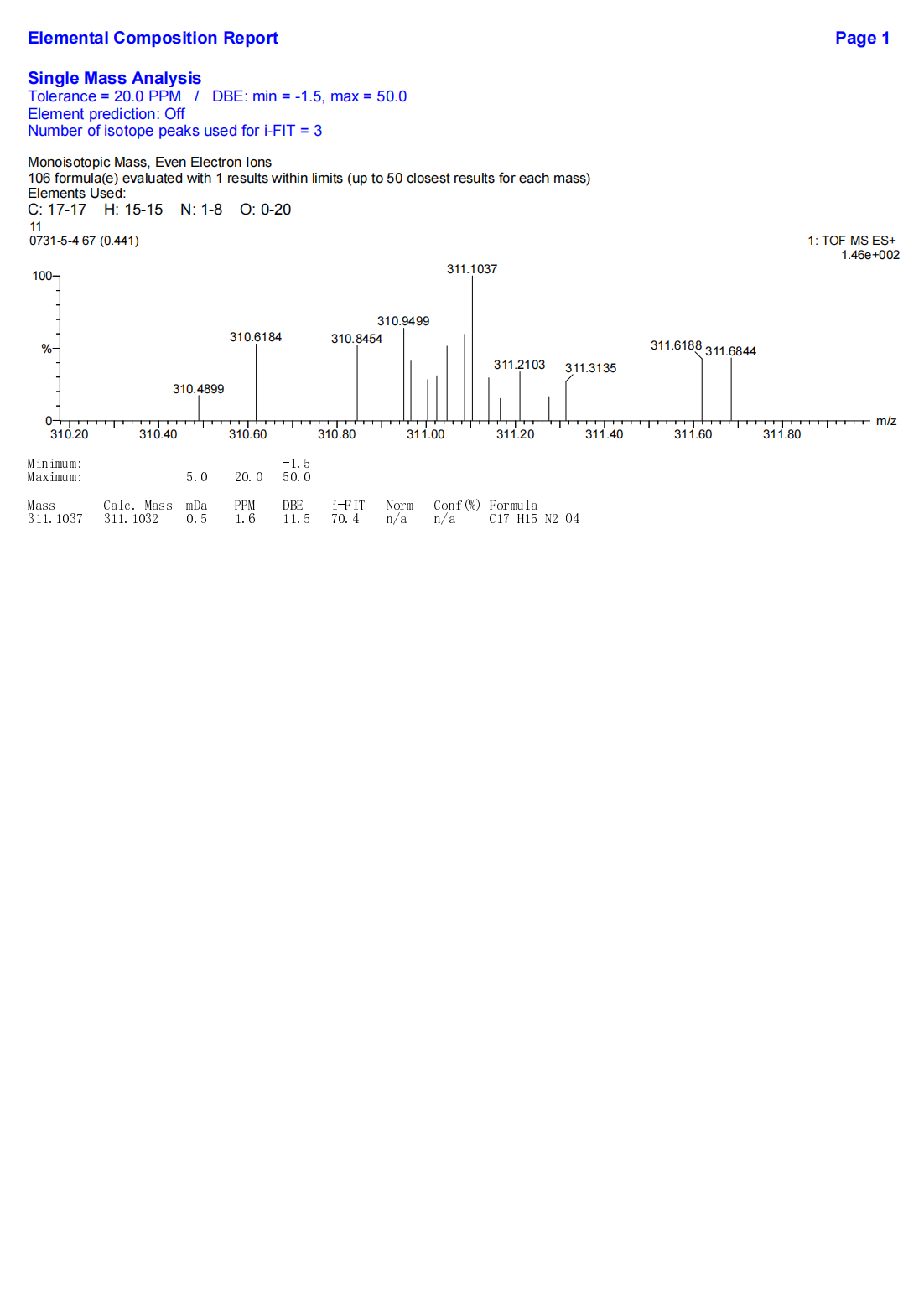


**Figure S13** HRESIMS spectrum for compound **4**

**Figure S14** UV spectrum for compound 4


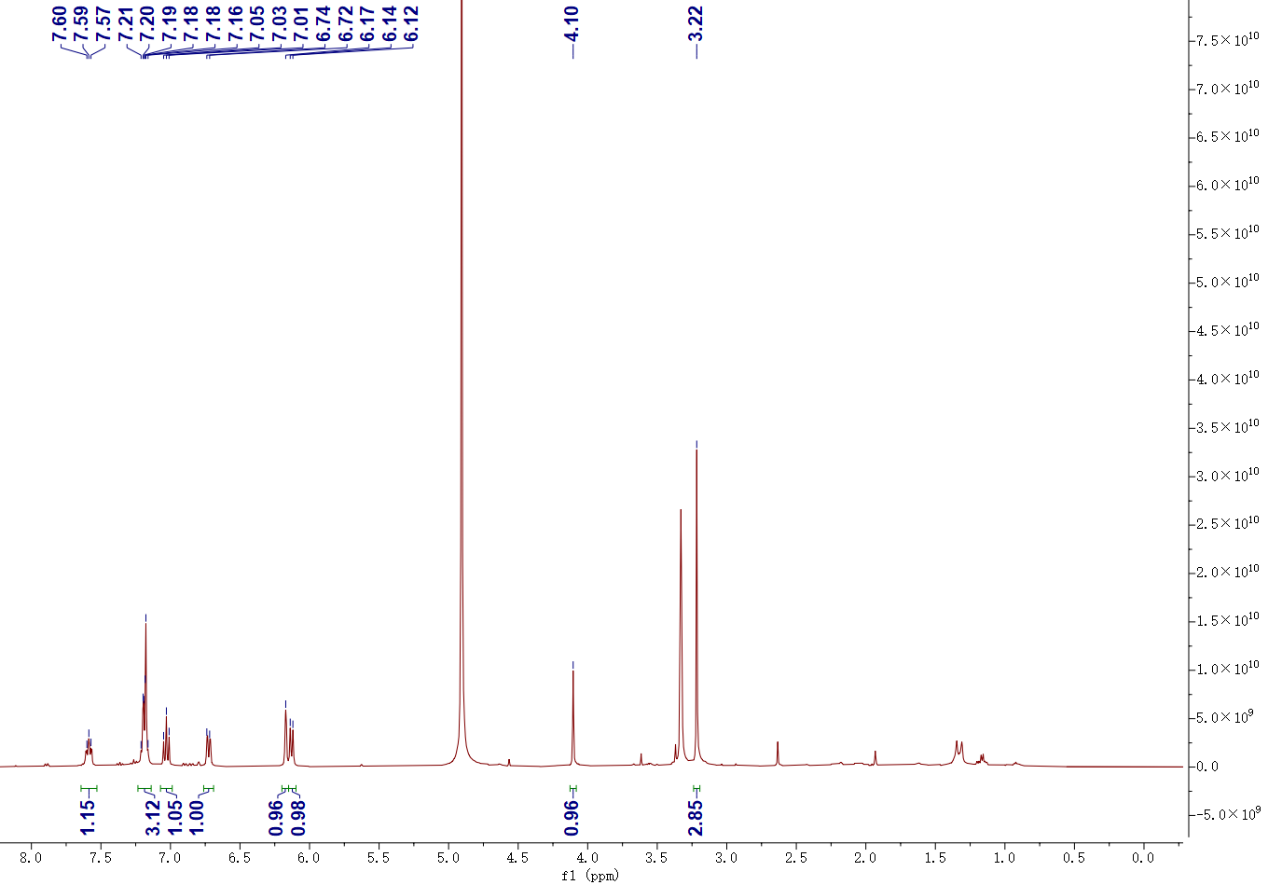


**Figure S15** ^1^H NMR spectrum for compound **4**


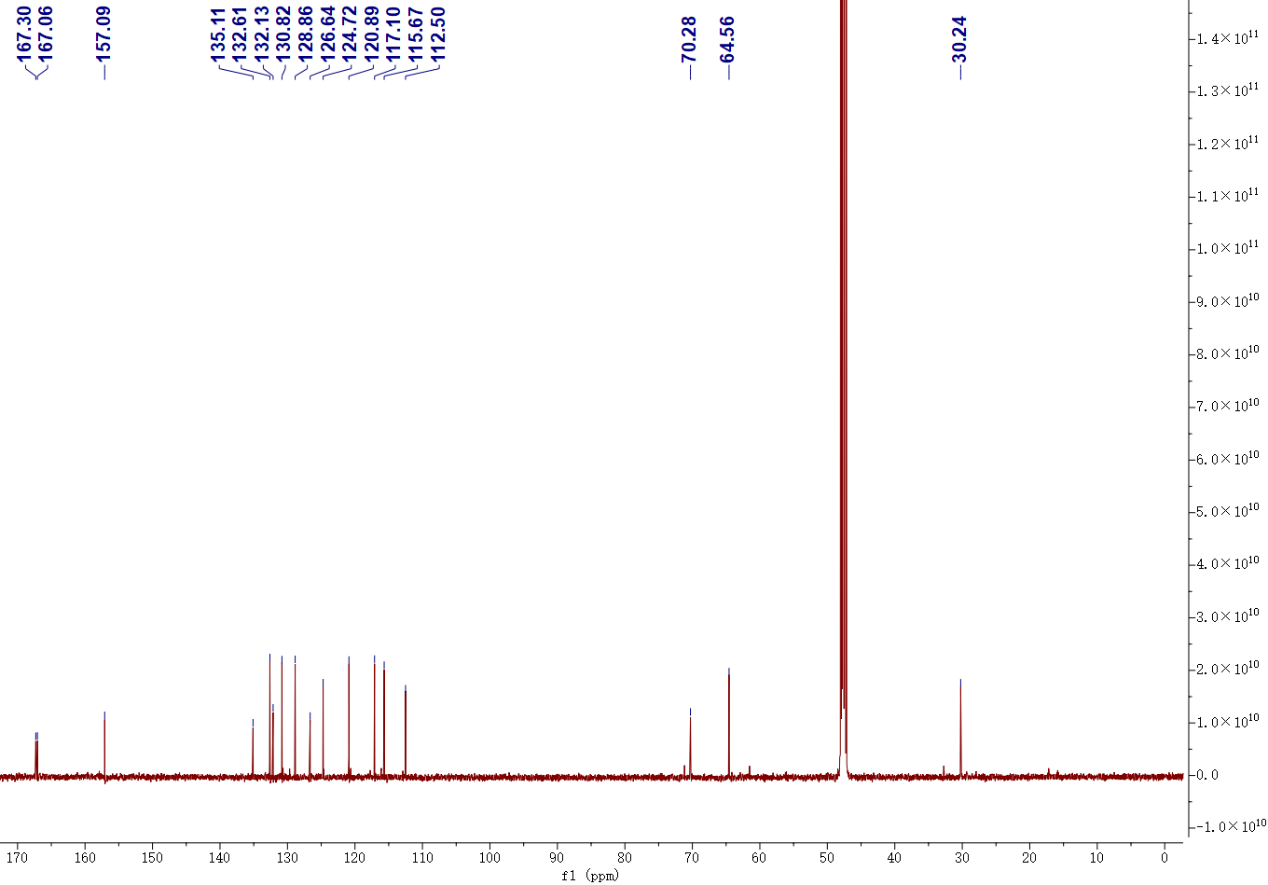


**Figure S16** ^13^C NMR spectrum for compound **4**


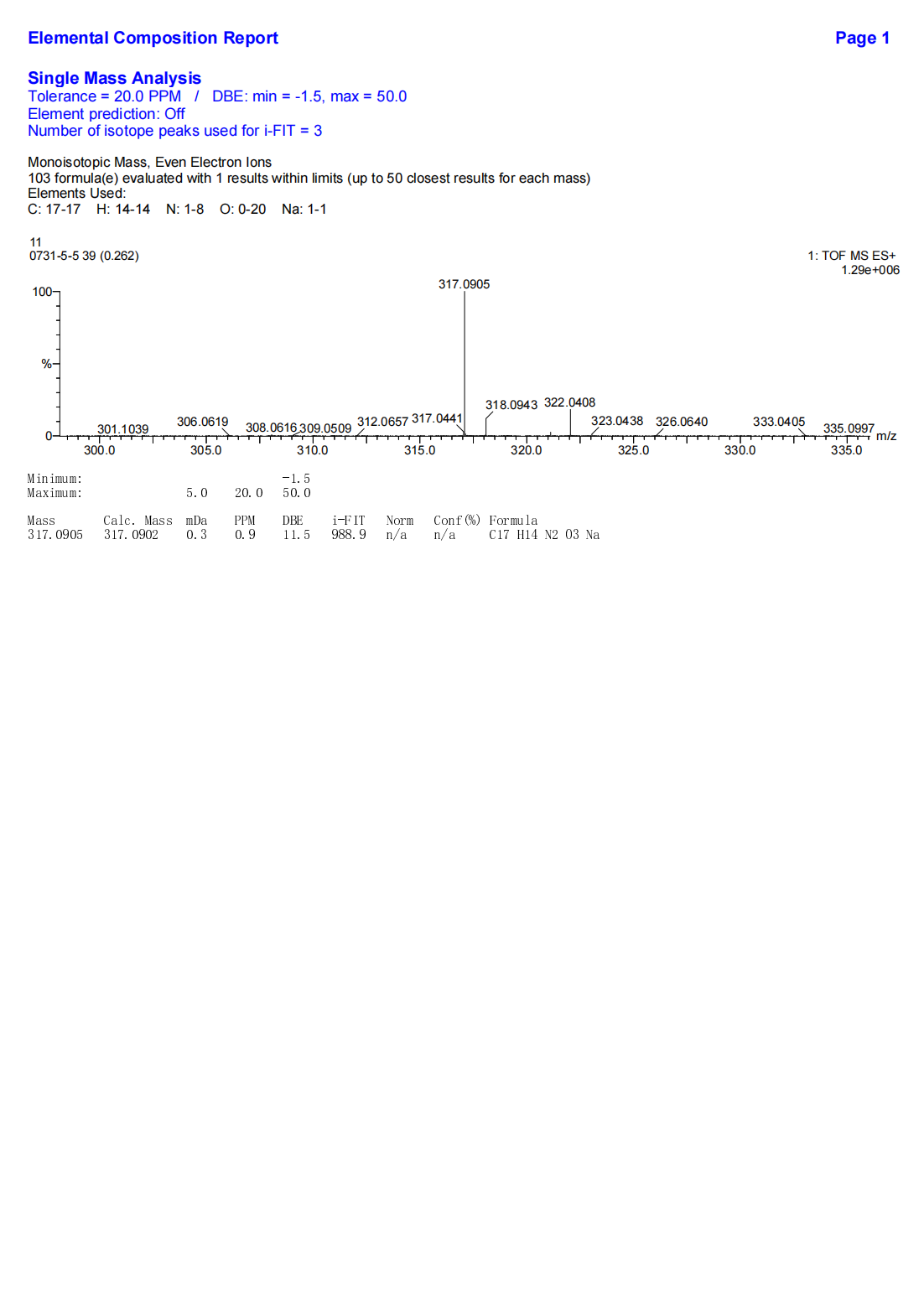


**Figure S17** HRESIMS spectrum for compound **5**

**Figure S18** UV spectrum for compound **5**


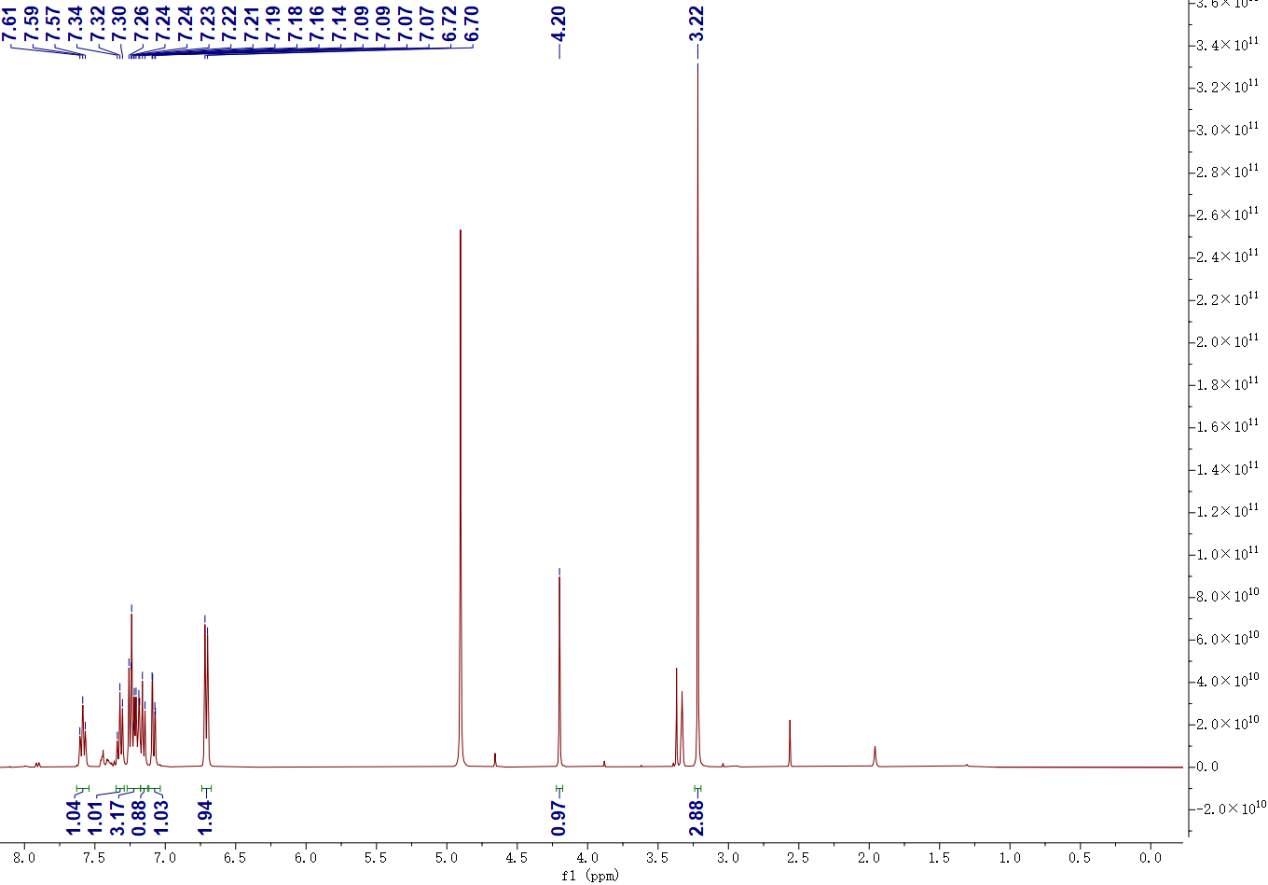


**Figure S19** ^1^H NMR spectrum for compound **5**


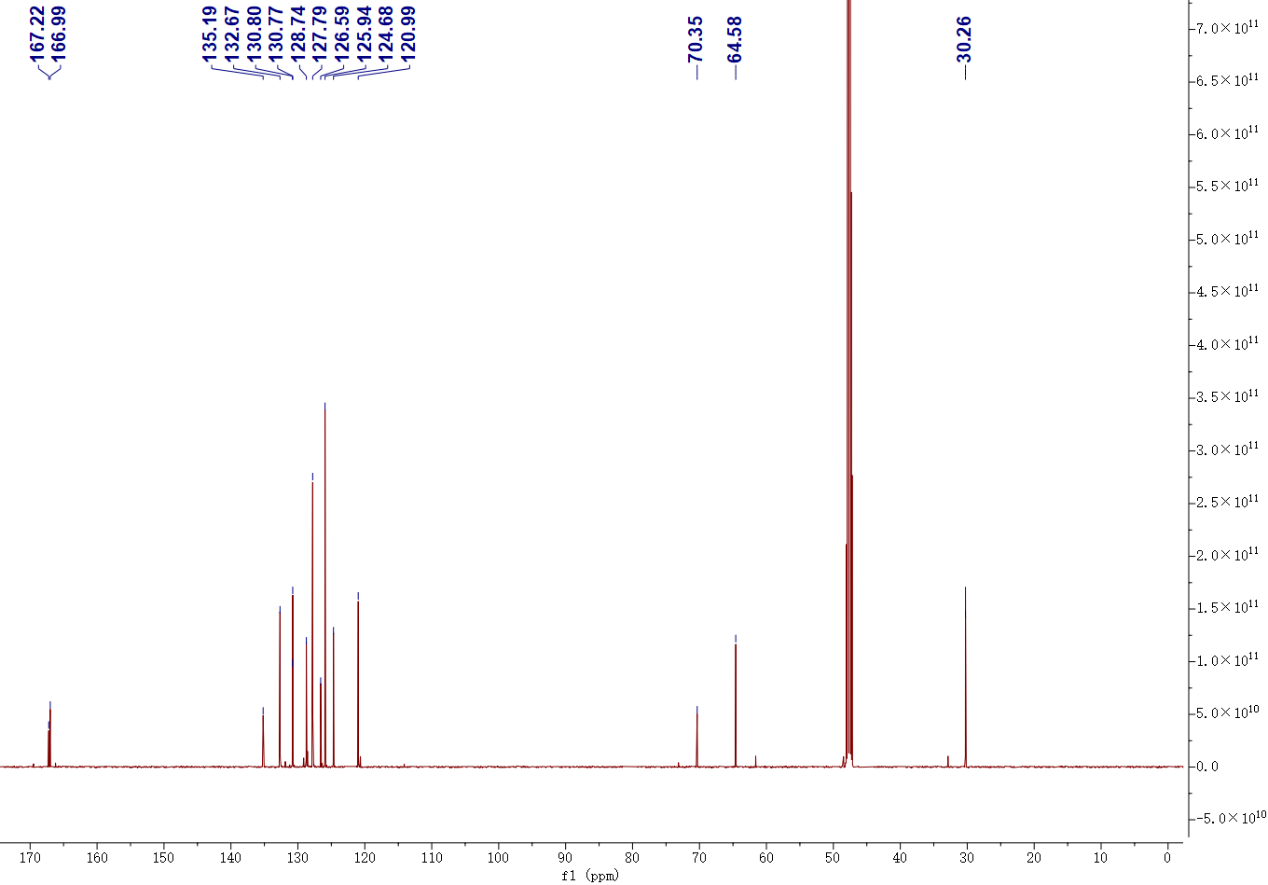


**Figure S20** ^13^C NMR spectrum for compound **5**


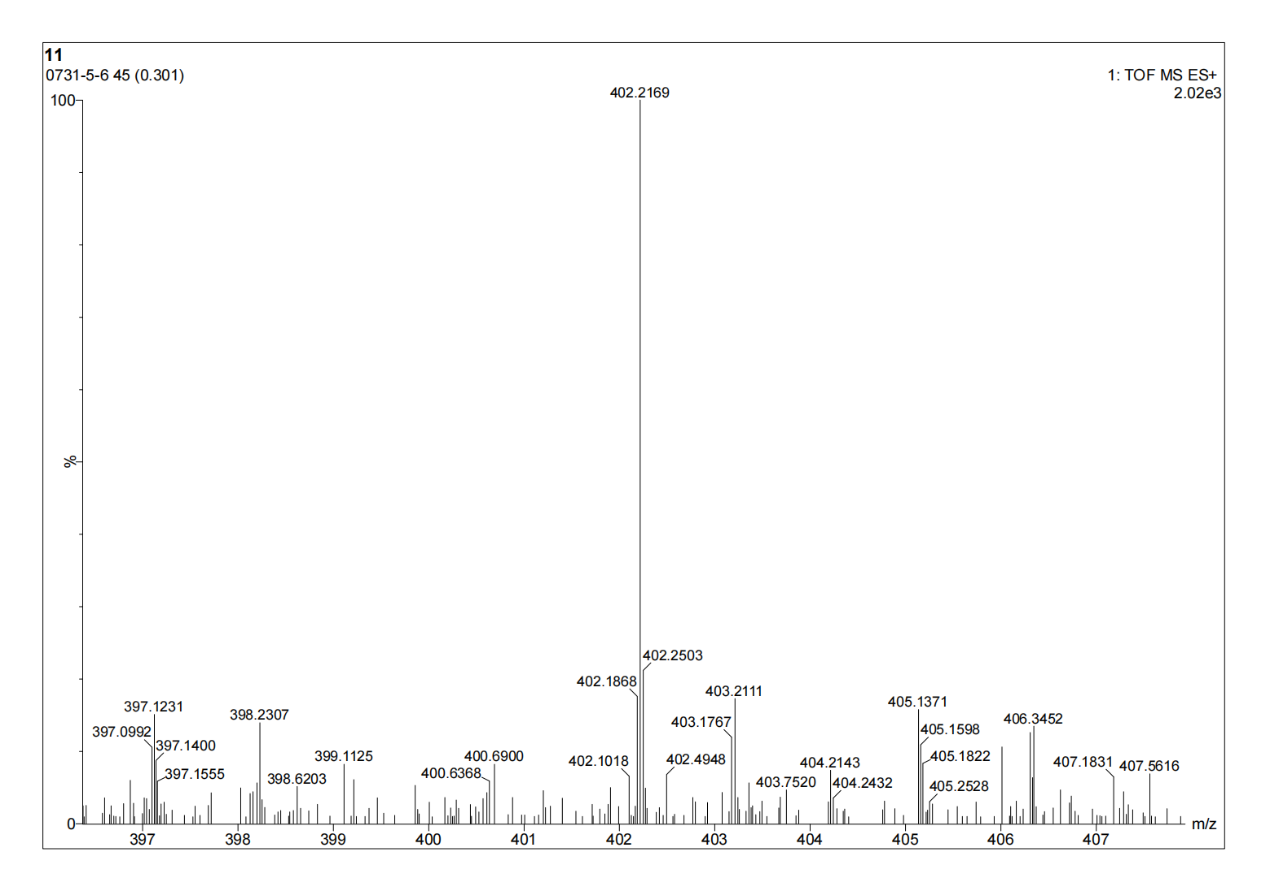

**Figure S21** HRESIMS spectrum for compound **6**

**Figure S22** UV spectrum for compound **6**


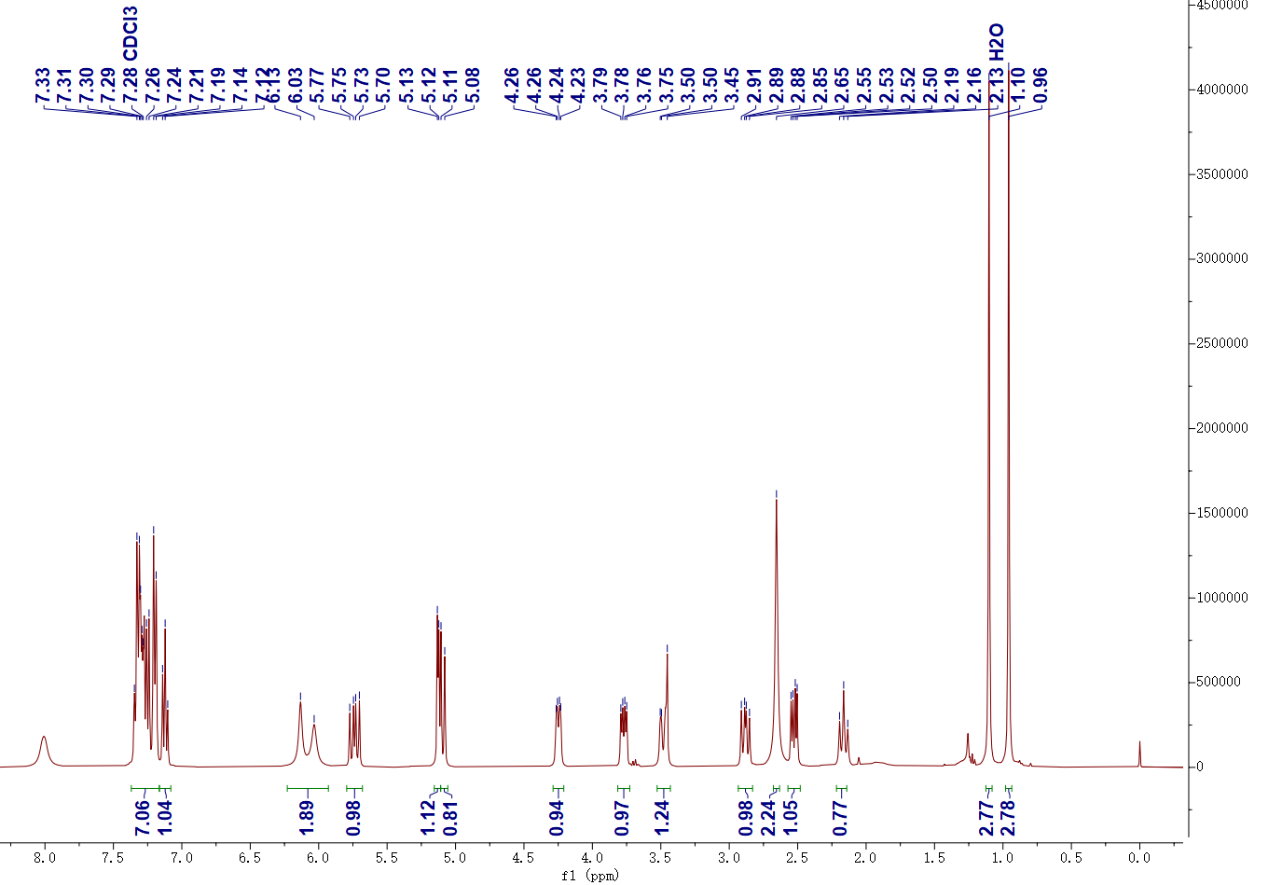


**Figure S23** ^1^H NMR spectrum for compound **6**


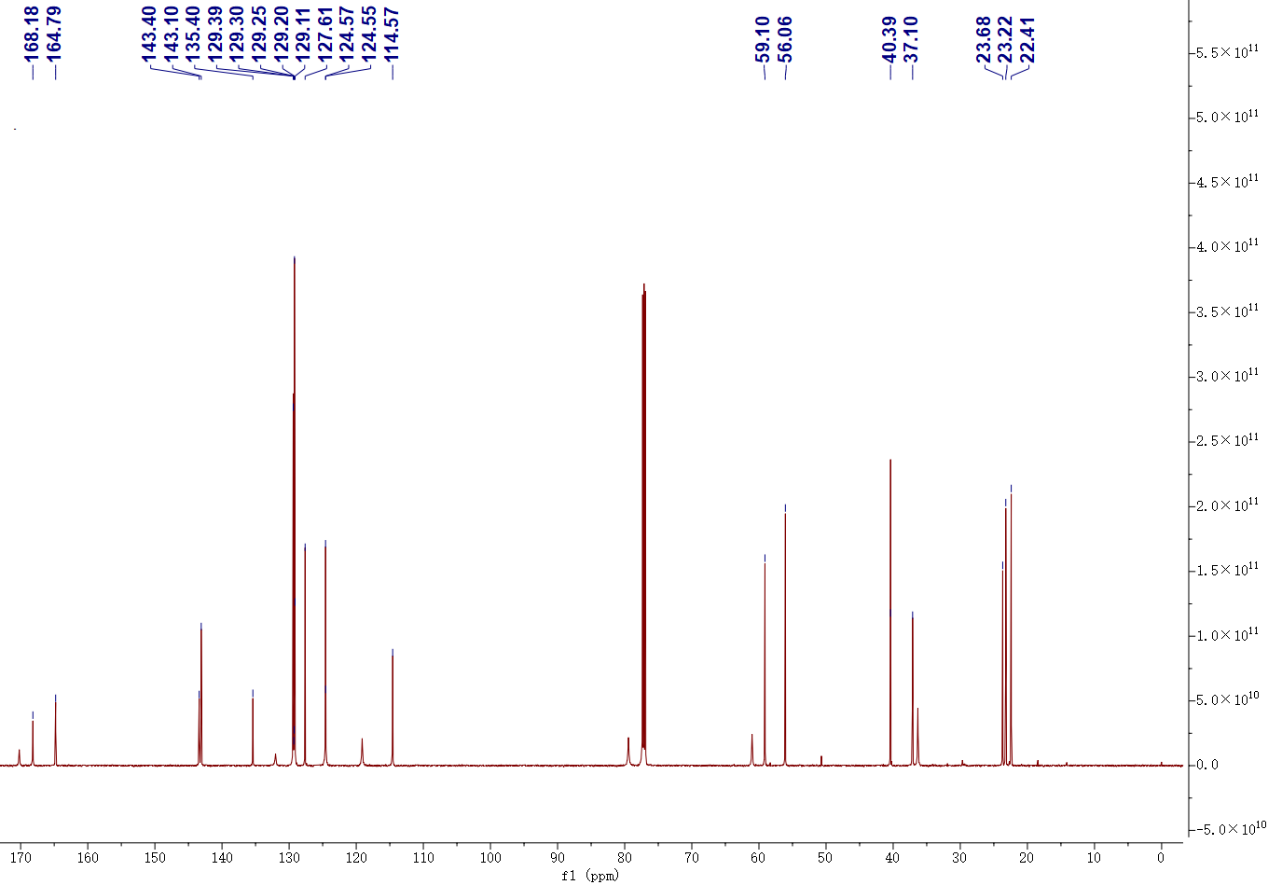


**Figure S24** ^13^C NMR spectrum for compound **6**


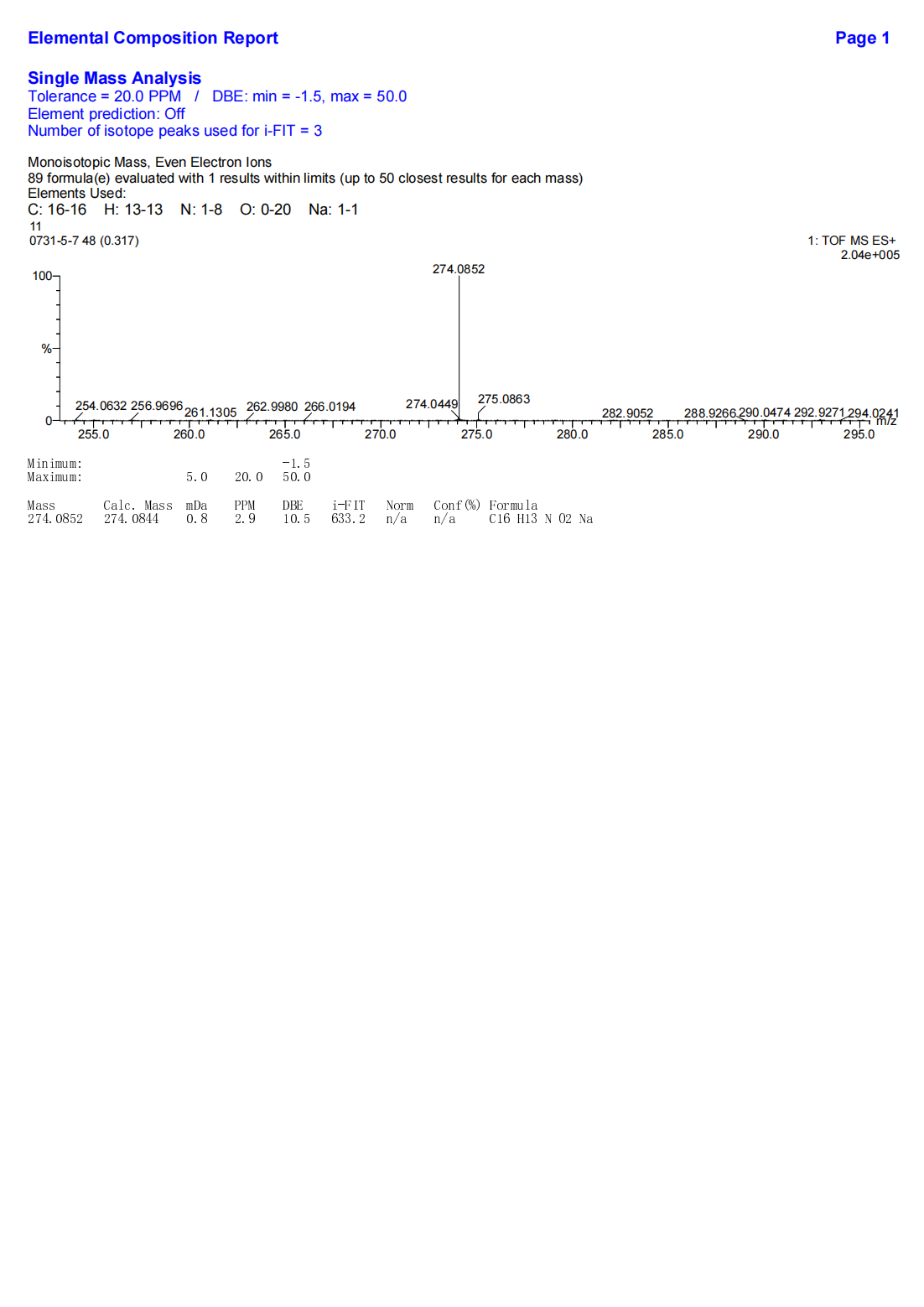


**Figure S25** HRESIMS spectrum for compound **7**

**Figure S26** UV spectrum for compound **7**


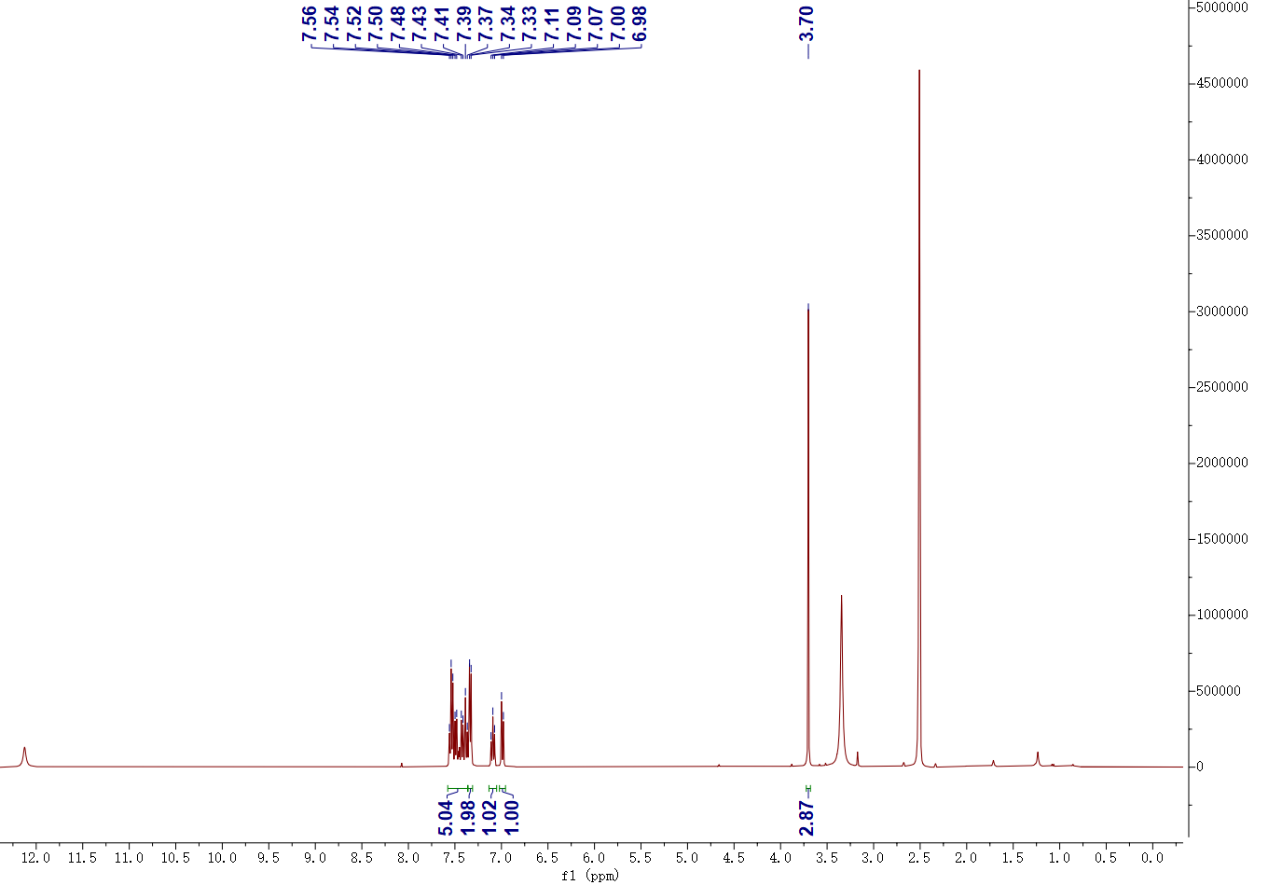


**Figure S27** ^1^H NMR spectrum for compound **7**


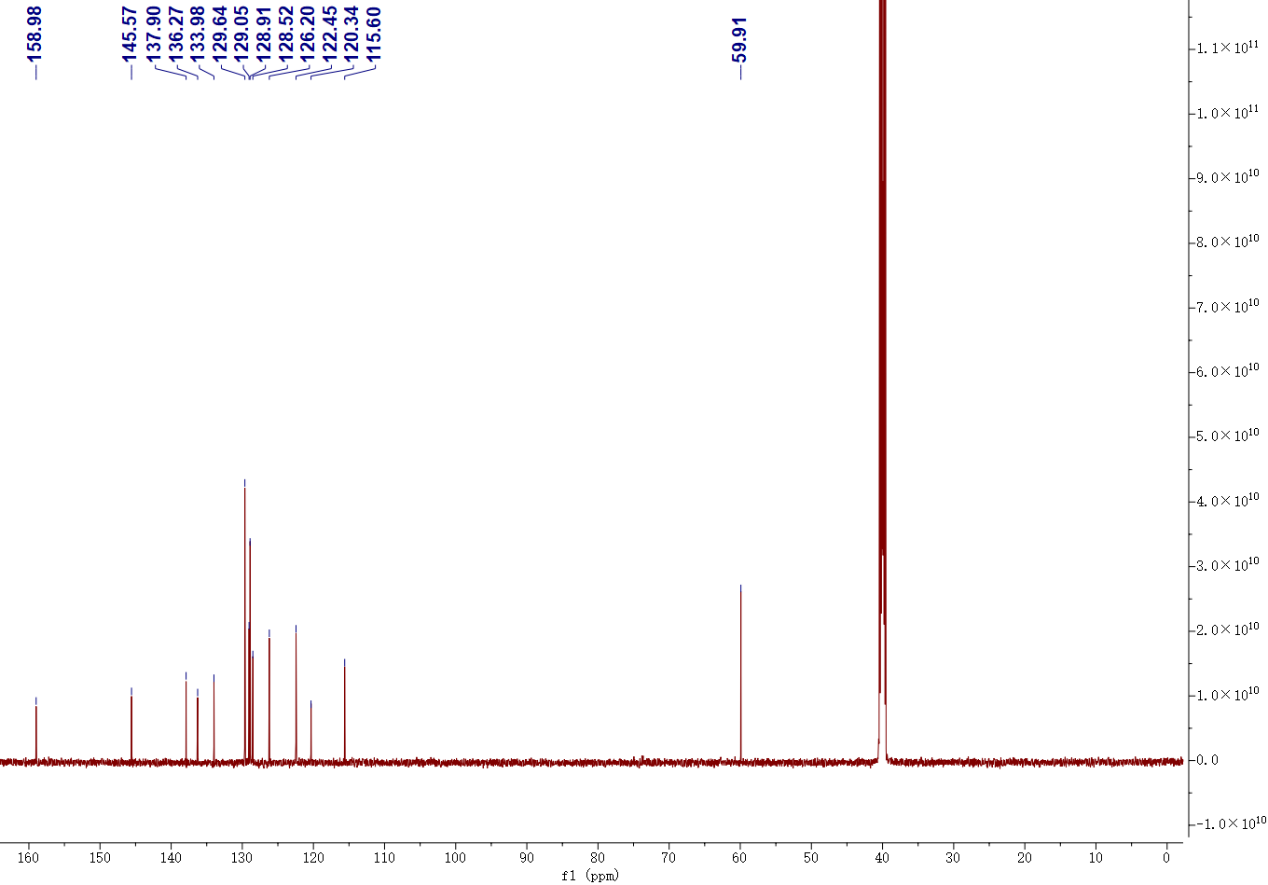


**Figure S28** ^13^C NMR spectrum for compound **7**
